# Biaxial Stretching of PBAT/PLA Blends for Improved Mechanical Properties

**DOI:** 10.3390/polym17192651

**Published:** 2025-09-30

**Authors:** Nikki Rodriguez, Osnat Gillor, Murat Guvendiren, Lisa Axe

**Affiliations:** 1 Otto H. York Chemical and Materials Engineering Department, New Jersey Institute of Technology, Newark, NJ 07102, USA; ncr22@njit.edu (N.R.); muratg@njit.edu (M.G.); 2 Zuckerberg Institute for Water Research, J. Blaustein Institutes for Desert Research, Ben Gurion University of the Negev, Midreshet Ben Gurion 8499000, Israel; gilloro@bgu.ac.il; 3 Department of Biomedical Engineering, New Jersey Institute of Technology, Newark, NJ 07102, USA

**Keywords:** biodegradable polymers, PLA, PBAT, biaxial stretching, films

## Abstract

Biodegradable polymers offer a promising solution to the growing issue of global microplastic pollution. To effectively replace conventional plastics, it is essential to develop strategies for tuning the properties of biodegradable polymers without relying on additives. Biaxial stretching promotes anisotropic crystallization in polymer domains, thereby altering the mechanical performance of polymer blends. In this study, we employed a design of experiment (DoE) approach to investigate the effects of biaxial stretching at three drawing temperatures (T_d_s) and draw ratios (λs) on a biodegradable blend of poly(lactic acid) (PLA) and poly(butylene adipate-*co*-terephthalate) (PBAT), aiming to optimize both the strength and ductility. The DoE analysis revealed that the composition, the λ, the interaction between the λ and composition, and the interaction between the T_d_ and composition significantly affect the elongation at break (ε_Break_). For the stress at break (σ_Break_), the most influential factors were the interaction between the λ and PLA concentration; a three-way interaction among the λ, PLA, and T_d_; the T_d_; the λ; and finally the PLA concentration alone. The optimal ε_Break_ and σ_Break_ were achieved at a λ = 5 × 5 and T_d_ = 110 °C, with a composition of 10% PLA and 90% PBAT. The stretched samples exhibited higher crystallinity compared to the pressed samples across all compositions. This work demonstrates that in addition to the composition, the processing parameters, such as the λ and T_d_, critically influence the mechanical properties, enabling performance enhancements without the need for compatibilizers or toxic additives.

## 1. Introduction

Poly(butylene adipate-*co*-terephthalate) (PBAT) and poly(lactic acid) (PLA) are two highly versatile biodegradable polymers with global applications in packaging, consumer goods, additive manufacturing, biomedical engineering, and agriculture [1]. As thermoplastics, PBAT and PLA can be thermally processed into functionalized geometries, blends, and/or composites at melt temperatures below their decomposition onsets. PBAT is a synthetic aliphatic–aromatic co-polyester developed in the 1990s and synthesized via polycondensation reactions from fossil fuel-sourced butanediol, adipic acid, and terephthalic acid [2]. PBAT consists of aromatic butylene terephthalate (BT) dimers and aliphatic butylene adipate (BA) dimers. BT dimers are random, rigid, and hydrolysis resistant, yet prone to crystallization. BA dimers are generally amorphous, flexible, and susceptible to hydrolysis and microbial degradation. Together, BT and BA yield a balanced polymer with poly(ethylene)-like ductility, making it particularly useful for flexible packaging and shopping bags [2,3]. PBAT also has excellent thermal stability with a low melting point of ~130 °C, so that it can be mechanically recycled up to seven times while retaining 90% of its pristine elastic modulus and tensile strength with or without compatibilizers [4]. On the other hand, PBAT has had limited success as a homopolymer due its relatively low strength (~21 MPa), high gas permeability [4], and relatively high production costs (high temperatures, long processing times) [3]. BASF is the largest manufacturer of commercially available PBAT, ecoflex^®^, with the standard grade being ecoflex^®^ F Blend C1200, made up of 55 mol% BT and 45 mol% BA [3,4]. According to the product’s specifications, ecoflex^®^ F Blend C1200 is certified industrially compostable and biodegradable via ASTM Standard D 6400-23 [5], European standards EN 13432 [6] and EN 17033 [7], Japanese standard GreenPla, and Australian standard AS 4736 [8,9].

Like PBAT, PLA is a semi-crystalline biodegradable polyester. Unlike PBAT, PLA is entirely aliphatic, strong, and brittle, making it useful for rigid packaging, textiles, electronics, medical devices, tissue engineering, and 3D printing [10]. PLA was first produced in the 1800s, although its commercialization did not advance until the 1990s [11]. In brief, PLA is produced from polymerization of lactide rings sourced from fermentation of starch-derived dextrose, making it a renewable and therefore highly desirable polymer [11]. The stereoisomerism of the lactide ring has implications for the tacticity of the resulting polymer chain, given that four stereoisomers are possible: L-lactide, D-lactide, a racemic mixture of L-lactide and D-lactide, and meso-lactide [11]. This stereoisomerism is introduced during the manufacturing process, where the fermentation processes preferentially produce the L-lactide precursor that result in L-units after polymerization [12]. PLA’s crystallizability is highly dependent on the molecular weight and the ratio of L- and D-monomers in its backbone [13,14]. D-lactic acid monomers introduce non-crystallizable co-monomer irregularities, leading to an exponential decrease in the crystallization rate with the D-monomer concentration [12]. In fact, PLA grades with >10% D-monomer concentrations are considered amorphous [12,14]. Blends of pure L-lactide monomer PLA (often abbreviated PLLA) and pure D-lactide monomer PLA (often abbreviated PDLA) can form interesting bulk crystalline phases with higher melting temperatures, but most commercial grades are co-block polymers with random D-monomers among a predominately L-monomer backbone [12]. A general comparison of PLA and PBAT is summarized in Table 1.

The polymer’s crystallinity is a critical parameter because it affects the gravimetric density, mechanical properties, and biodegradation rates [20]. Biaxial (orthogonal) or uniaxial stretching (also known as orientation processing) is used to induce crystallization, as exposure to high strain above a polymer’s *T_g_* accelerates crystallization kinetics [12]. The temperature at which a film is stretched is known as the draw temperature (T_d_). Biaxial stretching can be applied simultaneously in both directions or sequentially [23]. The draw ratio (λ) is the ratio of a film’s final length to its original length in either direction [23]. Both uniaxial and biaxial stretching induce changes in polymer crystallinity, with a greater λ yielding higher crystallinities [23]. NatureWorks LLC [24] reported that Ingeo^TM^ 4032D can be converted from a brittle film with a 6.0% ε_Break_ to a highly ductile film with an ε_Break_ between 100 and 180% via biaxial orientation processing [25]. Ingeo^TM^ 4032D has relatively low (approximately 1.4%) D-monomer concentrations and therefore low crystallinity, making it a good candidate for stretching and crystallization [13]. A number of techniques are available for laboratory-scale biaxial stretching, and include the Brueckner Karo^®^ equipment line, Labtech LP-S-50, rollers, custom-made systems, and pressure-induced flow processing [26]. Biaxial stretching of polymer blends or composites has precedent [15,17,18,27,28,29,30], but the field lacks a systematic understanding of the complex interplay between polymer composition(s), polymer grade(s), and overall stretching conditions. A polymer’s crystallizability is also influenced by the composition of the blend—as the constituents can have synergistic or antagonistic effects on crystallization. For example, Liu et al. [31] used Ingeo^TM^ 4032D and PBAT Ecoworld to show that biaxial stretching is not effective in the presence of compatibilizers like styrene–maleic anhydride copolymer. Gao et al. [18] explored the effect of λ (1 × 5 through 1 × 21.7) while uniaxially roll-drawing Ingeo^TM^ 4032D at 80 °C. They found that lower λs were more effective at increasing the ductility, with the largest increase occurring at λ of 1 × 7.5, with an increase in the ε_Break_ from 12.5% to 296% after drawing. Gao et al. [18] also found that the λ positively correlates with the σ_Break_, while it inversely correlates with the ε_Break_. Xu et al. [32] uniaxially stretched pure Ingeo^TM^ 4032D at 90 °C with λ of 1 × 5.3, increasing the ε_Break_ and σ_Break_ from 21% to 91% and 58 MPa to 130 MPa, respectively. Jariyasakoolroj et al. [15] used a Karo^®^ IV to stretch Ingeo^TM^ 4043D PLA, which has D-lactide concentration of 6%, to show that faster draw speeds (3–75 mm·s^−1^) generally result in larger increases in the ε_Break_ and σ_Break_, from 2.4–4.2% and 46.3–46.9 MPa to 11.7–77.9% and 46.9–174.0 MPa, respectively, depending on the stretching conditions [15]. Jariyasakoolroj et al. [15] attributed the larger ductility and strength after stretching to the smaller crystallite size, as the overall crystalline concentration alone (which plateaued at ~27%) could not explain the differences in behavior after 16 mm·s^−1^ for λ of 5 × 5 and 37 mm·s^−1^ for λ of 3 × 3.

Anisotropy is generally observed in industrial film processing techniques, such as blow film molding and subsequent rolling [18,33]; biaxial stretching is often used to study these commercial processes by reproducing similar temperatures, stretching speeds, and λs [26,34]. An understanding of the effects of processing on individual polymers is critical for obtaining reproducible, predictable, and optimal properties. Crystallization induced during processing depends on factors such as the duration, induced strain, and composition (e.g., polymer grade, additives, and nucleating agents). Chain extenders are lower-molecular-weight compounds with functional groups that bind polymer chains together, increasing both the molecular weight and interactions between polymer types. These compounds enhance miscibility and impact mechanical properties at concentrations as low as 0.5 wt% [35]. Chain extenders reduce polymer mobility, which in turn reduces crystallization rates [35]. The addition of chain extenders and compatibilizers also reduces the melt viscosity, which leads to less particle intercalation during composite mixing, and therefore worse processability [33]. Particle additives, such as talc or ZnO, in polymer matrices affect the bulk crystallization rates and mechanical properties depending on the particle composition, particle size, and polymer matrix [36,37]. For example, nanoparticles can lead to faster crystallization rates without significantly compromising the bulk properties at nucleating concentrations, as shown by Salaris et al. [37] for ZnO nanoparticle concentrations below 5 wt% in Ingeo^TM^ 3052D PLA matrix. The nucleating effect of solid particles was also evidenced by Shakoor et al. [36], who showed a reduction in the cold crystallization onset of PLA (Tate & Lyle grade HM1011, Mw 224,000 g/mol) from 127 °C to 96 °C when 900 nm talc particles were added at 10–20 wt%. Simultaneously, Shakoor et al. [36] found that ≥10 wt% of 900 nm talc particles significantly increased the PLA crystallinity (from 2% to up to 27% depending on the concentration), but also decreased the ε_Break_ (from 3.8% to 2–3% depending on the concentration) due to crack initiation at lower stresses within the polymer matrix. In summary, while particles can act as nucleating agents that increase crystallinity (leading to a mechanical advantage), they can also compromise a polymer matrix by introducing defects that exacerbate crack propagation (leading to an overall mechanical disadvantage). Therefore, achieving greater strength and ductility without the use of additives or compatibilizers is highly desirable. This is especially true for applications in agriculture or food packaging, where additives can be potentially toxic or have long-term negative consequences for the environment during biodegradation.

The design of experiment (DoE) approach statistically isolates effects [38]. The objective of our work is to elucidate the effects of biaxial stretching parameters and composition on the resulting thermal and mechanical properties, with an emphasis on optimizing the mechanical properties for a strong and ductile film. Moghaddam et al. [39] used a custom mixture DoE to optimize the mechanical properties (elastic modulus, ε_Break_, and impact strength) and equilibrium moisture concentrations of their PLA/poly(butylene succinate)/starch/wheat straw composites for application as single-use food container packaging. They were able to achieve the predicted properties for their optimized blend, with <7% relative deviation from their 23 experimental DoE runs [39]. Ortega et al. [26] utilized a Doehlert DoE with three levels to optimize the T_d_, stretch duration, and strain rate for Carbion Polymers PLA Luminy L105 grade. They characterized the film opacity, water absorption, melt flow index, chemical signatures via infrared spectroscopy, oscillatory rheology, mechanical tensile properties, and thermomechanical properties to optimize the mechanical properties [26]. Their results indicated that film processing did not significantly affect the water absorption or impact resistance, but affected the other mechanical properties [26]. Additionally, the temperature during biaxial stretching was a more significant factor than the stretching duration or strain rate [26]. Herein, we utilize a full factorial design DoE approach to investigate the roles and interactions of biaxial stretching and composition on the properties for a blend of PLA and PBAT.

## 2. Materials and Methods

### 2.1. Materials

Materials used include PBAT ecoflex^®^ F blend C1200 pellets (BASF, Ludwigshafen, Germany), PLA Ingeo^TM^ 4032D semi-crystalline high-heat film-grade pellets (NatureWorks LLC, Blaire, NE, USA), and ≥99.5% purity dichloromethane (DCM) (Sigma-Aldrich, St. Louis, MO, USA). All compositions are reported in mass percentage with sample weight measured via analytical scale (Fisher Scientific^TM^ Education ALF104, Hampton, NH, USA), with ±0.0001 g accuracy. Materials and samples were stored under vacuum or nitrogen glove box (Vacuum Technology Inc., Oak Ridge, TN, USA), with oxygen and water vapor concentrations less than 300 and 0.1 ppm, respectively.

### 2.2. Design of Experiment

A full factorial design of experiments was applied, with one center point and eight vertices for a total of nine runs (Table 2) using Minitab^®^ Statistical Software (Version 22.4.0). The factors included the PLA concentration (10–90 wt%)/PBAT concentration (90–10%), λ (2–5), and T_d_ (90–110°C). The range of temperatures was determined using the manufacturer specifications from NatureWorks LLC, with crystallization observed between 100 and 110 °C for Ingeo^TM^ 4032D [14]. The range of λ was selected based on earlier studies [15,17,18,27,28,29,30]. The stretching speed was fixed at 75 mm·s^−1^ based on Jariyasakoolroj et al.’s 2015 work [15], which demonstrated that 75 mm·s^−1^ is an optimal stretching speed for biaxially stretching a semi-crystalline grade of PLA with a comparable molecular weight. Control and validation runs were not used in the DoE analysis/regression but plotted for reference, and a 95% confidence interval was applied for variance analyses. The data collected were either part of the DoE design space (runs 1–9), control samples (runs 11–18), or reserved as validation data to evaluate the model’s robustness (runs 19–23) (Table 2) (see Supporting Information for regression analyses).

### 2.3. Film Preparation

Commercial feedstocks of polymer resin pellets (i.e., PBAT, PLA) were dissolved in DCM and blended using a process adapted from Yan et al. [40]. The DCM–polymer solutions were mixed to homogeneity then cast into a nonstick, thermally resistant poly (tetrafluoroethylene) cavity. The cast solution was dried in a vacuum oven (Yamato Scientific America Inc., Santa Clara, CA, USA, ADP31) under ambient pressure, then dried at 60 °C for 24 h, and finally dried under vacuum (≤0.08 atm) at 60 °C for an additional 24 h. Solvent blending of the polymer blend was applied due to logistics of equipment availability at the time, with the understanding that future work and larger batch sizes would require thermal mixing. The blended resins were cut into 1–2 mm square pieces and loaded into a bronze cavity (13 cm diameter, 0.033 cm height) for hot melt pressing. The samples were hydraulically pressed (Carver Laboratory Press, Wabash, IN, USA) at 0.84 MPa for 90 s (130 °C for pure PBAT and 170 °C for pure PLA and blends), following Lyu et al. [35]. After 90 s, the films were cooled to room temperature within 5 min at a rate of 1.5 °C·s^−1^ via cold water flow. The film thickness (after pressing and/or stretching) was measured using a Mitutoyo 7326A (Kawasaki City, Kanagawa, Japan) handheld dial thickness gauge with ±0.005 mm accuracy, or a Model 6066 tabletop dial gage (Chicago Dial Indicator Co., Des Plaines, IL, USA) with ± 0.00254 mm accuracy.

### 2.4. Biaxial Stretching

Biaxial stretching was performed on a Brückner Karo^®^ V (Brueckner Group USA, Dover, NH, USA). A square sample of 9 cm × 9 cm was loaded, clamped, and then stretched. The thickness was measured for each film prior to and after stretching. The λ was symmetrical in both the machine direction (MD) and the transverse direction (TD); these were labeled on each sample after stretching for use during mechanical testing. The samples were biaxially stretched simultaneously for 30 s at 75 mm·s^−1^, then annealed for 10 s at 90 °C, 100 °C, or 110 °C before release. The ovens were pre-heated to 90 °C, 100 °C, or 110 °C prior to stretching, with the clip temperature set to 80 °C for 90 °C processing and 85 °C for 100 °C or 110 °C processing, with the fans set to 50% throughout the process.

### 2.5. Mechanical Testing

Mechanical testing was performed on an MTS Criterion Model 43 (MTS Systems Corporation, Eden Prairie, MN, USA) fitted with coarse sand-blasted grips or an Instron^®^ 5967 (Instron^®^, Norwood, MA, USA) fitted with tapered grips. ASTM Standard D 638-22 Type V dog-bones were cut with ASTM-certified cutting dies (Fremont Cutting Dies, Inc., Fremont, OH, USA). The ε_Break_, σ_Break_, and elastic modulus (E) were characterized fivefold for each sample from the engineering stress–strain curves obtained at a 50 mm·min^−1^ testing speed and room temperature. There was no designation of MD or TD for the hot melt-pressed (not stretched) control samples because the technique is isotropic, but the samples were cut along the same direction for all the replicates. All the stretched samples were characterized in the MD only. The ε_Break_ and σ_Break_ were statistically analyzed using analysis of variance (ANOVA), polynomial regression, and Tukey’s Honestly Significant Difference (HSD) test for pairwise mean comparisons with 95% confidence (see Supporting Information).

### 2.6. Differential Scanning Calorimetry (DSC)

The thermal phase behavior and degree of PLA crystallinity (Χ_PLA_) were evaluated using differential scanning calorimetry (DSC) performed with a DSC 6000 (PerkinElmer Inc., Shelton, CT, USA) with 20 mL·min^−1^ nitrogen gas flow. The samples were crimp-sealed in 50 µL vented aluminum pans with 2–9 mg sample loading per run. The temperature program was as follows: isotherm for 1.0 min at 30 °C, heating from 30 °C to 200 °C at 10 °C·min^−1^, isotherm for 3.0 min at 200 °C, cooling from 200 °C to −60 °C at 10 °C·min^−1^, and heating from −60 °C to 200 °C at 10 °C·min^−1^. The enthalpies obtained from the first heating ramp were used to calculate the X_PLA_ [35,41,42,43,44]. The first heating ramp represents the crystallinity due to processing (i.e., stretching), while the second heating ramp represents the crystallinity due to composition (e.g., nucleating effects), as the first heating/cooling cycle essentially erases the material’s thermal history. The following equation was used:

(1) $${Χ}_{PLA}=\frac{{∆H}_{m}-{∆H}_{cc}}{m\cdot {∆H}_{fusion}^{0}}\cdot 100\%$$

where ΔH_m_ is the measured heat of fusion of the blend, ΔH_cc_ is the measured cold crystallization peak during heating, m is the mass fraction of the polymer of interest in the blend (if applicable), and ${∆H}_{fusion}^{0}$ is the standard heat of fusion for a pure infinitely thick crystalline form [45]. The value of 107 J·g^−1^, published by Righetti et al., 2015 [46], was selected as the $\;{∆H}_{fusion}^{0}$ of PLA in our work due to evidence of α’ crystals [46,47]. The ΔH_cc_ was set to zero when absent or indistinguishable from other peaks. The glass transition temperature (*T_g_*) was identified as the inflection point between the change in heat capacities circa 62 °C for each sample using built-in software.

### 2.7. Thermogravimetric Analysis (TGA)

The temperatures at 10% mass loss (90% remaining mass, abbreviated T_90_), 15% mass loss (85% remaining mass, abbreviated T_85_), 50% mass loss (50% remaining mass, abbreviated T_50_), residual solvent, and char yield (remaining sample weight at 600 °C) were evaluated using thermogravimetric analysis (TGA) on a TGA 8000 (PerkinElmer Inc., Waltham, MA, USA). The T_85_ was interpreted as the onset of decomposition as predicated by Velásquez et al., 2019 [48]. The samples were loaded into the platinum pan with approximately 10 mg sample loading per run. The temperature program was performed under 20 mL·min^−1^ nitrogen gas flow as follows: isotherm for 5 min at 30 °C followed by heating from 30 °C to 600 °C at 10 °C·min^−1^.

### 2.8. Scanning Electron Microscopy (SEM)

Particle morphology was observed using JEOL JSM-7900F (JEOL USA Inc., Peabody, MA, USA) field emission scanning electron microscope (SEM). Samples were coated with 9.9 nm 80Au-20Pd to minimize charging. Images were taken with secondary electron detector at 0.5–5.0 kV. SEM was performed on four samples: pressed 10%/90% PLA/PBAT (17 ^C^), 90%/10% PLA/PBAT λ 5 × 5 90 °C (2 ^D^), 50%/50% PLA/PBAT λ 5 × 5 90 °C (7 ^D^), and pure PLA λ 5 × 5 90 °C (11 ^D^).

### 2.9. X-Ray Diffraction (XRD)

The XRD was characterized using a PANalytical Empyrean (Malvern Panalytical Ltd., Almelo, The Netherlands) X-ray diffractometer with a 27 mm monocrystalline Si zero-background sample holder. The operating conditions involved 45 kV and 40 mA using Cu Kα radiation (λ = 1.5438 Å). Crystalline concentration (X) of each blend was calculated using the following equation:

(2) $$X=\frac{I_{crystalline}}{I_{amorphous}+I_{crystalline}}\cdot 100$$

where I_crystalline_ and I_amorphous_ are the areas under the crystalline and amorphous signals of the XRD diffractograms [35]. Note that the instrument background signal (“constant background signal”) was subtracted from all diffractograms prior to calculation of X. The crystallize size (D) was calculated using the Scherrer equation below:

(3) $$D=\frac{K\cdot \lambda }{\beta \cdot cos\theta }$$

where β is the peak’s full width at half maximum (FWHM) in radians; λ = 0.15438 nm; θ is the Bragg angle (peak position) in radians; and K is the shape factor, assumed to be 0.9 for PLA and PBAT [49].

## 3. Results and Discussion

### 3.1. Mechanical Properties

**Figure 1:**
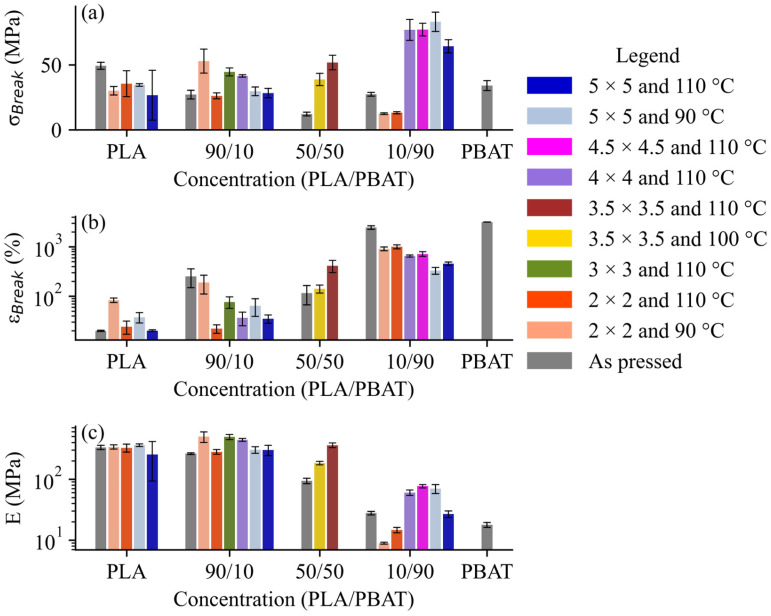
Average (n = 5) mechanical properties ± standard error (n = 5) organized by composition for all runs. Mechanical properties include (**a**) σ_Break_, (**b**) ε_Break_, and (**c**) E. Pairwise significance results between each run using Tukey’s Honestly Significant Difference (HSD) test can be found in Supporting Information.

Mechanical properties are critical in food packaging, agriculture mulch, biomedical engineering, and shipping material. The tensile test results illustrate the impact of biaxial stretching conditions on the mechanical properties, ε_Break_, σ_Break_, and E, of the samples, with the unstretched controls represented in grey (Figure 1; see Supporting Information for full mechanical stress–strain curves).

The mechanical behavior of PLA/PBAT blends under various stretching conditions was evaluated through the ε_Break_, σ_Break_, and E, as shown in Figure 1 and Table 3. The stretched samples with as little as 10% PLA exhibited changes in their mechanical properties (Figure 1, Supporting Information). The engineering stress–strain curves revealed necking and strain hardening in our samples, consistent with other studies [15,17,18,27,28,29,30,50] (see Supporting Information). The highly ductile samples (e.g., samples with 10% PLA) exhibited a gradual transition from elastic to plastic behavior with strain hardening. The region used to calculate the E was the relatively narrow linear region at the start of the experiment, after which the stress–strain curve became more curved but did not clearly abruptly yield. This behavior is typical for polymers [51]. The E for our samples ranged from 8.9 MPa to 495.8 MPa and was positively correlated with the PLA concentration (Figure 1). Wang et al. [27] characterized the E of PLA Ingeo^TM^ 4032D injection molded at 200 °C as 1438.5 ± 56.6 MPa, which was much higher than our value of 330.9 ± 58.7 MPa. Wang et al. [27] characterized the E of 90% PLA (Ingeo^TM^ 4032D) and 10% PBAT (TH801) injection molded at 200 °C as 1293.7 ± 73.0 MPa, which was also much higher than ours of 261.3 ± 18.2 MPa. Chen et al. [28] calculated Es of 1162.5 ± 169 MPa and 1575 ± 113 MPa for pure PLA Ingeo^TM^ 4032D. Upon further inspection, it is likely that these discrepancies are due to calculating the strain using the grip separation (i.e., 25.4 mm) instead of the gage length (i.e., 7.62 mm). Using the gage length, as recommended by ASTM Standard D 638-22 [51], would result in E values of 7.62/25.4x, the Es obtained using the nominal strain. A high PLA concentration, low T_d_, and higher λ favor the production of materials that are stiff (high E), have a high σ_Break_, and are brittle (low ε_Break_), while the reverse conditions yield soft, extensible materials. The improved mechanical performance of blends with increased PBAT concentrations after stretching (Figure 1) was also unexpected, given that biaxial stretching enhances crystalline domains of PLA at a faster rate [31]. Interestingly, although the NatureWorks LLC specification sheet [25] lists the ε_Break_ and σ_Break_ for a 4032D film to be 100–180% and 103–145 MPa, respectively, for a film biaxially stretched to a λ of 3.5 × 5 (temperature not specified), we were not able to achieve this strength and ductility for any of our 100% PLA films. The largest increase in ductility (19.9% to 83.5% after stretching) was seen in the 100% PLA after stretching to a λ of 2 × 2 at 90 °C. The general trend from our results is that most stretching conditions enhance the σ_Break_ at the expense of ε_Break_ (Figure 1, Supporting Information). Stretching enhances the molecular alignment, resulting in stronger materials with less entanglement and extensibility before fracture (ductility) [15,18]. Our work shows that the relationship between processing and mechanical properties is complex, because in two cases, runs 3 ^D^ (10%/90% PLA/PBAT, λ 2 × 2, and T_d_ 110 °C) and 4 ^D^ (10%/90% PLA/PBAT, λ 2 × 2, and T_d_ 90 °C), the samples experienced a clear reduction in both the ε_Break_ and σ_Break_, although the reduction in the σ_Break_ was statistically insignificant (Figure 1; see Supporting Information for statistical analyses). Still, the significant reduction in the ε_Break_ without a statistically significant change in the σ_Break_ compared to the unstretched 10%/90% PLA/PBAT control (run 17 ^C^) reflects a complex relationship between the polymer microstructure and resulting mechanical properties. Studies [28,31] demonstrated that, in some cases, biaxial stretching enhances both mechanical properties [28], while in others there was a tradeoff between strength and ductility [31]. Jariyasakoolroj et al. [15] used 2D wide-angle X-ray diffraction to study the microstructure of Ingeo^TM^ 4043D PLA after chill roll casting a film followed by biaxially stretching it to λ of 3 × 3 or 5 × 5 on a Karo^®^ IV [15]. They found that the PLA *crystallite size* was inversely related to both the σ_Break_ and ε_Break_ [15]. Overall, the smallest crystallites occurred at a stretching of 75 mm s^−1^, 5 × 5, and 90 °C [15]. These conditions are equivalent to our run 11 ^C^, except for using a PLA grade (Ingeo^TM^ 4043D) with different D-monomer concentrations. Specifically, the D-monomer concentration of 4043D was 6% compared to 1.4% for 4032D, and the Mw/Mn for 4043D and 4032D were 166 kDa/116 kDa and 106 kDa/223 kDa, respectively [15,18]. Therefore, the isotropic nano-scale crystallites reduced the PLA’s brittleness, resulting in stretched samples with larger crystallite domains [15]. Chen et al. [28] used a heated (90 °C) roller to produce a film with λ of 1 × 5.3, increasing the ε_Break_ from 21% to 106% and the σ_Break_ from 46 MPa to 117 MPa [28]. Our results highlight that the mechanical properties of a material can be manipulated with biaxial stretching protocols, but achieving an optimal balance between strength and ductility requires careful selection of processing conditions.

### 3.2. Thermal Properties

**Figure 2:**
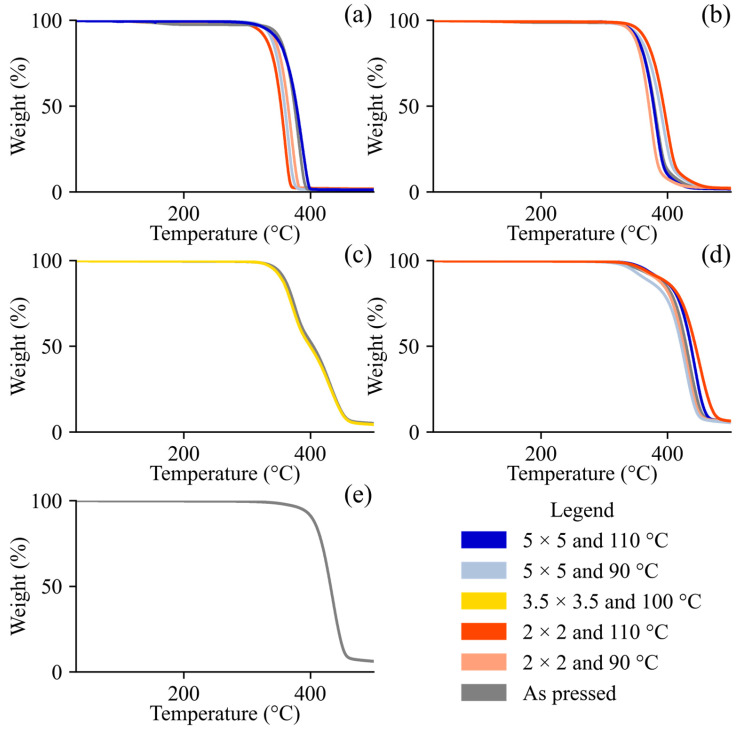
TGA thermograms of DoE runs (1 ^D^, 2 ^D^, 3 ^D^, 4 ^D^, 5 ^D^, 6 ^D^, 7 ^D^, 8 ^D^ and 9 ^D^) and control runs (10 ^C^, 11 ^C^, 12 ^C^, 13 ^C^, 14 ^C^, 15 ^C^, 16 ^C^, 17 ^C^, and 18 ^C^) organized by composition: (**a**) pure PLA, (**b**) 90%/10% PLA/PBAT, (**c**) 50%/50% PLA/PBAT, (**d**) 10%/90% PLA/PBAT, and (**e**) pure PBAT.

**Figure 3:**
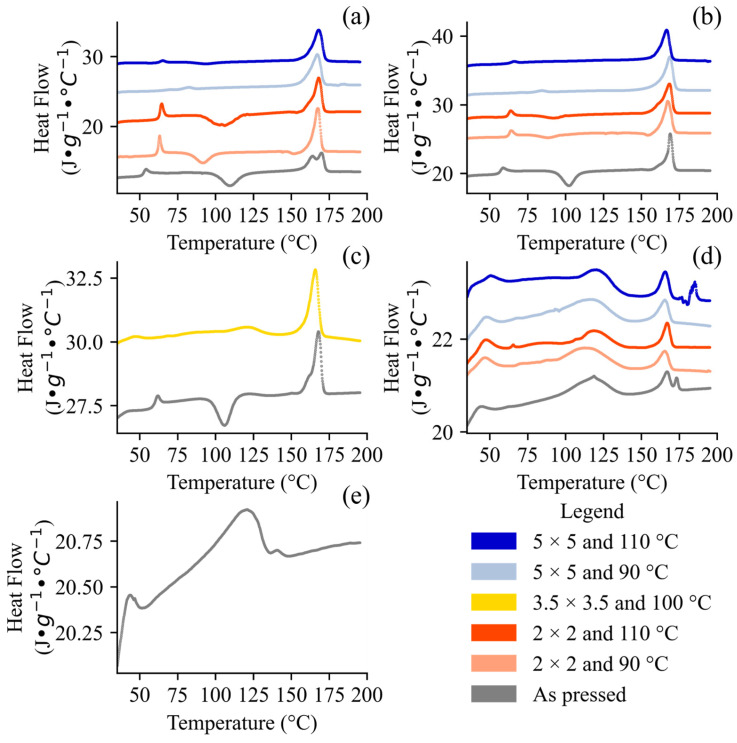
DSC thermograms, Endo up, for the first heating scans. These scans depict the DoE runs (1 ^D^, 2 ^D^, 3 ^D^, 4 ^D^, 5 ^D^, 6 ^D^, 7 ^D^, 8 ^D^, and 9 ^D^) and control runs (10 ^C^, 11 ^C^, 12 ^C^, 13 ^C^, 14 ^C^, 15 ^C^, 16 ^C^, 17 ^C^, and 18 ^C^) organized by composition: (**a**) pure PLA, (**b**) 90%/10% PLA/PBAT, (**c**) 50%/50% PLA/PBAT, (**d**) 10%/90% PLA/PBAT, and (**e**) pure PBAT.

**Figure 4:**
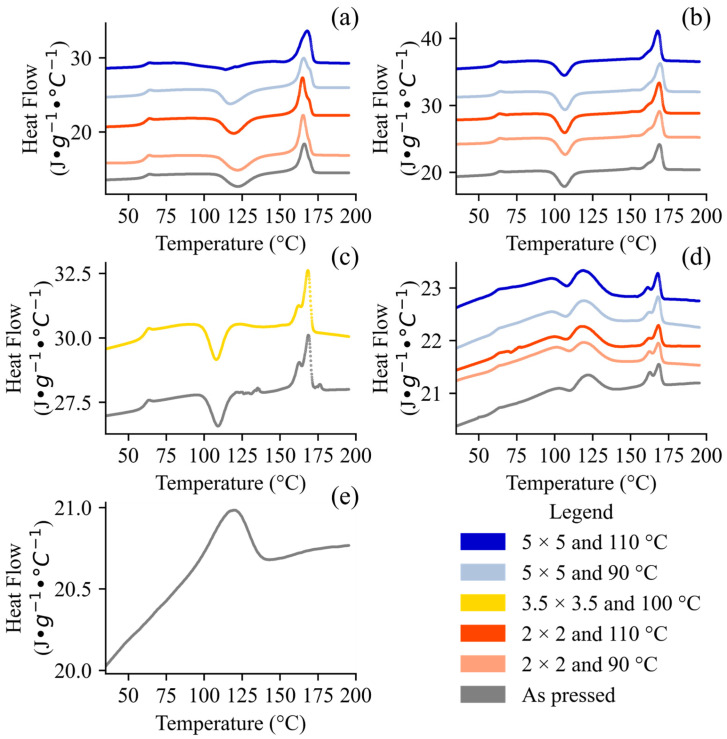
DSC thermograms, Endo up, for second heating scans. These scans depict DoE runs (1 ^D^, 2 ^D^, 3 ^D^, 4 ^D^, 5 ^D^, 6 ^D^, 7 ^D^, 8 ^D^, and 9 ^D^) and control runs (10 ^C^, 11 ^C^, 12 ^C^, 13 ^C^, 14 ^C^, 15 ^C^, 16 ^C^, 17 ^C^, and 18 ^C^) organized by composition: (**a**) pure PLA, (**b**) 90%/10% PLA/PBAT, (**c**) 50%/50% PLA/PBAT, (**d**) 10%/90% PLA/PBAT, and (**e**) pure PBAT.

The thermal properties provide insight into the crystallinity and thermal stability processing of and/or composition imparted to our samples. The TGA results for the DoE and control samples are presented in Figure 2. All the samples exhibited a primary degradation event, marked by a sharp mass loss, which is characteristic of polymer decomposition [52]. In general, the PLA and PBAT exhibited distinct decomposition temperatures around 340 °C and 410 °C, respectively [52] (Figure 2). Our average T_85_ for pure PLA (347.2 °C ± 23.2 °C), irrespective of stretching conditions, is in agreement with what has been reported in the literature for the same grade. Zhu et al. [53] reported 334 °C while Takkalkar et al. [54] reported 338 °C (melt pressed) and 337 °C (solvent casted) using both PLA Ingeo^TM^ 4032D and 10 °C·min^−1^. Interestingly, our T_85_ for pure PBAT (409.5 °C) deviates more from the values of the same grade reported by Raffaela de Matos Costa et al. [55] (387 °C), who used a heating rate of 20 °C·min^−1^, and Lyu et al. [35] (339 °C), who used a heating rate of 10 °C·min^−1^. Run 18 ^C^ yielded a similar result when repeated with a lower sample loading (9.417 mg vs. 15.507 mg) to ensure that the results were not shifted due to the larger samples having a delayed heat transfer. A similar phenomenon occurs with higher heating rates, as higher heating rates result in lower heat transfer efficiency [56], but the most common heating rate found in the literature, 10 °C·min^−1^, was selected for our studies. One possible explanation for both polymers exhibiting delayed thermal decomposition relative to the literature is the age and storage of the polymers prior to characterization. Fu et al. [20] and Reit et al. [57] showed that as PLA and/or PBAT age or are intentionally biodegraded, their TGA thermal decomposition behavior shifts to lower temperatures. PLA/PBAT blends exhibit bimodal degradation behaviors, with biaxial stretching appearing to shift these two peaks depending on the stretching conditions. In some cases, such as for run 5 ^D^ (50%/50% PLA/PBAT, λ = 3.5 × 3.5, and 100 °C), stretching did not affect the thermal stability. The onset of degradation (T_85_), T_90_, and T_50_ were strongly negatively correlated with the PLA concentration, but weakly correlated with the stretching parameters (λ, T_d_) (Figure 6). The T_85_ ranged between 335.11 °C and 409.52 °C, with the PLA clearly decomposing earlier than the PBAT in its pure form and in blends. The pure samples (pure PBAT or pure PLA) had narrower decomposition phenomena than the blends. For example, the average range between the T_90_ and T_50_ was 25 °C for pure PLA and 29 °C for pure PBAT. For the blends, this range was higher at 31 °C for 90%/10% PLA/PBAT, 47 °C for 50%/50% PLA/PBAT, and 52 °C for 10%/90% PLA/PBAT. All the samples were observed to be thermally stable below 110 °C for all compositions shown in Figure 2, indicating that processing did not induce thermal degradation. The Figure 2 results also reveal minimal weight changes at low temperatures (i.e., <100 °C), demonstrating that the samples were sufficiently dried with no residual water/solvent prior to characterization [58]. The thermal analyses via DSC also revealed qualitative signatures of PBAT crystallinity and quantitative signatures of PLA crystallinity. The results from the first and second heating scans are presented in Figure 3 and Figure 4, respectively.

The distinct thermal properties of the first (Figure 3) and second (Figure 4) heating scans alone is evidence of the effect of processing on our samples. First, the very broad and less defined PBAT melting peaks reflect its lower crystallinity owing to its irregular BA/BT structure. Kaoudom et al. [59] stretched pure ecoflex^®^ F Blend C1200 PBAT and found drastic changes in the mechanical properties, indicating that while PBAT has a slower crystallization rate than PLA [60], it is not negligible. This effect may have been a confounding factor in our films, as we were unable to resolve the crystalline concentration of PBAT due to overlapping peaks and the broad melting of the distinct BT/BA segments. Characterization of PBAT crystallinity using DSC has generally not been reported for PLA/PBAT blends; in most cases, only the PLA crystallinity has been characterized [29,61,62,63,64]. Sun et al. [42] studied PBAT/PLA blends with a PBAT/PLA ratio of 9/1 aged outdoors between 1 and 4 months and found that the PBAT crystallinity (characterized using the first heating scan of DSC) ranged between 10.32 and 11.4%, and was greater than the PLA crystallinity, which varied from 2.4 to 2.7%. In our samples with at least 50% PBAT, the crystalline PBAT peaks were well defined, with strong BT segments (around 45 °C) irrespective of their thermal histories (Figure 4). Although we expected this peak to decrease the PLA *T_g_* due to some plasticization via PBAT, the peak remained in the hot melt-pressed samples for all the drawn samples (the first heating scan but not the second one) and even in a thermogram of pristine resin from the manufacturer (see Supporting Information). This result indicates that the PBAT exhibited some degree of crystallinity not introduced during biaxial stretching (see Supporting Information), which re-crystallized too slowly to be captured in the second heating scan (Figure 4).

The DSC thermograms (Figure 3 and Figure 4) and Equation (1) were used to quantify the Χ_PLA_ for each sample (Table 3). The first heating scan reflects the thermal history, while the second indicates the compositional effects. The PLA melting peaks ranged between 138 and 172 °C depending on the thermal history, molecular weight, and monomer composition, but the role of thermal history is not well established [15,16,18,29,30,36,64,65]. The wide temperature range and broad melting behavior was due in part to PLA’s various polymorphs (α, α’, β, γ), as well as mesophases of highly oriented non-crystalline polymer chains, which have unique bulk thermal properties [46]. For a given grade of PLA, its β melting point is about 10 °C less than that of the α, which is the most stable polymorph [12]. For example, Echeverria et al. [66] found that highly oriented PLA, produced by electrospinning, exhibited lower decomposition temperatures, which they attributed to the formation of the thermodynamically less stable β form being predisposed to lower degradation. In our own samples, we saw a clear shift in the melting behavior after stretching. The stretched samples exhibited a generally monomodal melting phenomenon that began at 160 °C and peaked at 167 °C, while the pressed samples experienced a bimodal melting phenomenon with two peaks around 163 °C and then again between 168 and 173 °C (Figure 4). In addition, the *T_g_s* of the stretched samples were 3–10 °C greater than that of the pressed precursor, consistent with formation of a mesophase—that is slightly more thermodynamically favorable than a completely disordered amorphous phase (Figure 3). Zheng et al. [67], who uniaxially stretched pure PLA Ingeo^TM^ 4032D at 70 °C using a uniaxial tensile tester, also found that the *T_g_* increased with the λ. The biaxially stretched samples with at least 90% PLA exhibited interesting changes in their glass transition behavior. For the samples with λ of 2 × 2 (irrespective of temperature), the first heating scan showed a strong enthalpic relaxation (ΔH_r_) after the change in heat capacity that accompanies a glass transition (Figure 3a–d), but this phenomenon was absent in the second heating scan (Figure 4a–d). An endothermic transition after a glass transition, ΔH_r_, is characteristic of amorphous (including mesophases) materials [30,68]. The ΔH_r_ intensity is dependent on a material’s thermal history, aging, and other properties [68]. For our samples, given that they were stored under a similar temperature and were of the same grade, we can attribute their differences to processing. Amorphous materials quickly relax into a more stable, organized configuration above their *T_g_*, which yields a visible endotherm [68]. Ouchiar et al. [30] found that the mesophase concentration was higher for a larger λ only when the samples were stretched at low temperatures (i.e., 70 °C), while the mesophase concentration was absent or very low for samples stretched at 90 °C, irrespective of λ. Consulting the literature shows that PLA properties are highly grade- and process-sensitive, because Zheng et al. [67], who stretched PLA Ingeo^TM^ 4032D uniaxially, and Ouchiar et al. [30], who stretched NaturePlast PLE003 biaxially, stretched their samples at the same T_d_ (70 °C) and comparable λs (1 × 2 or 2 × 2 and 1 × 3 or 3 × 3), but obtained markedly different DSC thermograms, with no visible ΔH_r_ in that of Zheng et al. [67]. For our own samples, we expected the hot melt-pressed samples and those stretched at a low λ (e.g., 2 × 2) to exhibit a larger ΔH_r_ than the samples with greater crystallinity; this trend was generally true (Figure 3), although the highly stretched samples still showed evidence of PLA mesophase [68,69]. It is possible that with higher heating rates this overlap could have been avoided, because increased rates reduce crystallization during heating [68]. For compositions with >10% PLA, cold crystallization peaks were absent or very minor after stretching (Figure 3) but reappeared in the second heating scan (Figure 4), indicating that the effect was a product of the material’s thermal history. The disappearance of cold crystallization peaks due to stretching was also established by Zheng et al. [67] for the same grade of PLA. Cold crystallization was also present in the hot melt-pressed (“As pressed”) samples with >10% PLA during both the first (Figure 3) and second (Figure 4) heating scans (see Supporting Information for validation dataset results). Most of the melting behaviors converged and became indistinguishable from their pressed precursor sheets in the second heating scan (Figure 4), with a bimodal melting behavior that likely reflected the PLA polymorphs produced during the DSC heating and cooling cycle.

Biaxial stretching induced an increase in the Χ_PLA_ irrespective of processing conditions in all cases except one of biaxial stretching of PLA composites [15,17,18,27,28,29,30]. The only exception was reported in a study with NaturePlast PLA grade PLE003 stretched at 90 °C and λ of 2 × 2, which did not experience any change in crystallinity based on DSC and wide-angle X-ray scattering [30]. The crystalline fractions detected with DSC after stretching have varied widely, between 1.0 and 70.9% [15,17,18,27,28,29,30]. Our results, summarized in Table 3, are consistent with the effect of higher λs yielding greater crystallinity concentrations [15,17,18,27,28,29,30]. The *T_g_* and cold crystallization peaks are properties of the amorphous regions of the polymer, while the well-defined melting peaks are properties of the crystalline regions, which are essentially immobilized compared to the amorphous regions [68]. The pressed pure PLA exhibited cold crystallization in the first heating run, while the biaxially stretched samples did not, as shown in Figure 3. This observation is indicative of crystallinity induced by stretching. The absence of cold crystallization peaks for the biaxially stretched samples mirrors that of Simmons et al. [70], who annealed PLA in the presence of nucleating agents or compatibilizers at 180, 100, or 120 °C. We observed this behavior in the biaxially oriented samples because of the long-range ordered mesophase, a precursor to the crystalline phase, which then reorganizes above the *T_g_* [15]. On the other hand, mesophases generally accompany cold crystallization peaks and therefore the origin of this behavior is unclear [66]. The PLA mesophase is more ductile (an order of magnitude larger) than purely amorphous PLA, and as a result quantification of the Χ_PLA_ solely via DSC may not be able to account for all the changes in mechanical properties [69].

A polymer is “plasticized” when its molecular mobility is increased, which is accompanied by a reduction in the *T_g_* [71]. This behavior is observed with the addition of a plasticizer that increases the molecular mobility of the polymer in question [71]. Because the *T_g_* is a property of the amorphous portion of the polymer, it is possible that the crystallinity is too great to produce a detectable signal. Interestingly, Zheng et al. [67] found that beyond λ of 2.0, the *T_g_* was no longer detectable via DSC but was detectable using a dynamic mechanical analyzer (DMA). The *T_g_* for our material, when present, is consistent with that found in the literature for the same grade [72]. Indeed, the addition of plasticizers has been shown to reduce the glass transition temperature of PLA down to as low as 37 °C, but still above room temperature [72]. Furthermore, the melting temperature for the crystalline fractions was reduced by approximately 8–9 °C [72]. Interestingly, the *T_g_* was very subtle or even absent for some samples, especially those with less than 50% PLA (Table 3, Figure 3). PBAT and PLA are largely regarded as immiscible polymers, which is evidenced by the distinct *T_g_*s when blended. A miscible blend would have a *T_g_* at an intermediate temperature [68]. Although a decrease in the *T_g_* is possible, the *T_g_* in these cases was not visible even at lower temperatures (Figure 3), and for most samples it reappeared in the second heating scan (Figure 4), indicating it was a product of processing.

Luo et al., 2019 [73], found that as the molecular weight of PLA Ingeo^TM^ 4032D decreases due to biodegradation during composting, the bimodal melting behavior of PLA crystalline peaks reduces to the lower-temperature monomodal peak. This phenomenon may be due to the amorphous portions degrading at an accelerated rate compared to the crystalline fractions, with the sample therefore exhibiting a large signal from its crystalline fraction. Overall, the first heating scan of our films indicated that the biaxially stretched films exhibited a monomodal melting peak that changed to a bimodal one during the second heating (Figure 4). The change from monomodal crystalline behavior (Figure 3) to the more defined, bimodal melting (Figure 4) exhibited in our work parallels that during degradation, where the distribution of crystallites changes. In the case of our materials, the change in distribution was due to the homogenous crystal structure induced by biaxial stretching (first heating) becoming a mixture of small and large crystal domains during the second heating due to the melting and cooling and re-melting of the sample in the DSC.

### 3.3. X-Ray Diffraction

**Figure 5:**
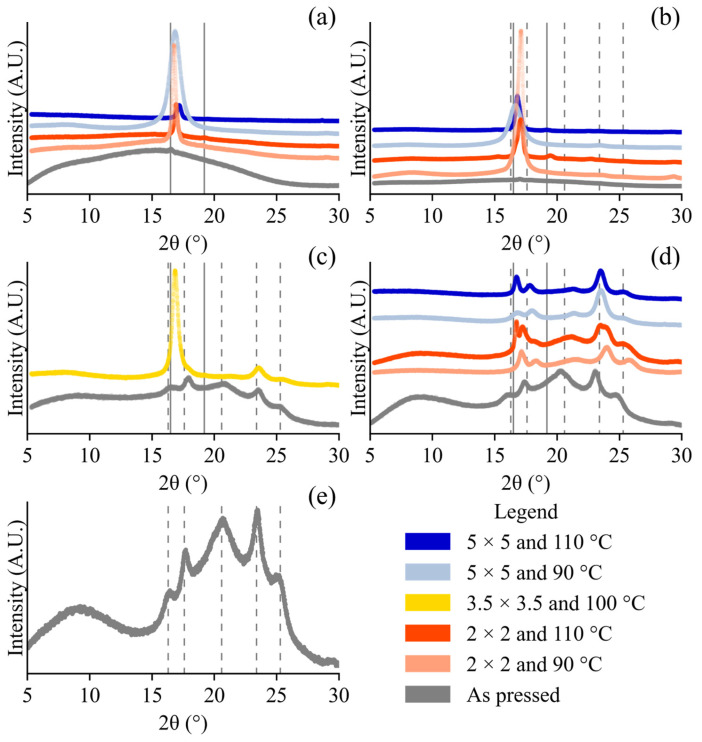
XRD traces for DoE runs (1 ^D^, 2 ^D^, 3 ^D^, 4 ^D^, 5 ^D^, 6 ^D^, 7 ^D^, 8 ^D^, and 9 ^D^) and control runs (10 ^C^, 11 ^C^, 12 ^C^, 13 ^C^, 14 ^C^, 15 ^C^, 16 ^C^, 17 ^C^, and 18 ^C^) organized by composition: (**a**) pure PLA, (**b**) 90%/10% PLA/PBAT, (**c**) 50%/50% PLA/PBAT, (**d**) 10%/90% PLA/PBAT, and (**e**) pure PBAT. Solid lines represent phases attributed to as-pressed PLA (2θ 16.5°, 19.2°), while dashed lines represent phases for as-pressed PBAT (2θ 16.3°, 17.6°, 20.6°, 23.4°, 25.3°).

The XRD diffractograms (Figure 5) and Equation (2) were used to characterize the film crystallinity of PLA and PBAT. The PLA exhibited one very prominent peak at 2θ of 16.516°, and subtler peaks at 14.9–15.1°, 19.1–19.2°, 22.3–22.6°, 25.2°, and 28.3–29.0° 2θ (Figure 5). For the samples with ≥90% PLA, the 2θ 16.516° peak was narrower for λ of 2 × 2 than λ of 5 × 5 (irrespective of T_d_). The intensity of this peak was also greater at 90 °C than 110 °C (irrespective of λ). PLA Ingeo^TM^ 4032D α and α’ have very similar peaks at 2θ 16.4–16.6° and 18.5–19.0° [46,73,74]. PLA β is less common, less prominent, and has not been characterized for the specific grade of PLA we used, to the best of our knowledge. PLA β polymorph has been characterized using another grade of PLA, with peaks at 25.8°, 26.5°, and 28.1° 2θ [66]. However, these peaks have also been attributed to the α phase in other studies [46]. PLA α or β peaks were apparent in some of our samples, although the signals were relatively small compared to the well-defined α or α‘ peak at a 2θ of 16.5° (Figure 5). We expect that our material contains more α’ than α phase because α contains many more peaks along with a higher melting peak of 180 °C (Figure 3). Unfortunately, using XRD, we were not able to resolve the differences in the signals between the mesophase and amorphous phases [75]. PBAT, which has slower crystallization kinetics, has a broadly amorphous signal, with the sharpest crystal diffraction peaks at 2θ 16.227°, 17.649°, 20.617°, 23.423°, and 25.269° (Figure 5) [76,77]. These peaks shifted subtly and varied in their relative intensity depending on the stretching conditions (Figure 5) [76,77]. Peak shifts indicate changes in interplanar spacing of polymer systems [78]. Shifts to a higher 2θ indicate a denser packing structure for the PLA and PBAT phases, which occurred in all the stretched samples with respect to their as-pressed counterparts (Figure 5). The broad peaks (such as the one circa 2θ ≈ 8°) were attributed to PBAT’s amorphous halo, as observed by others using the same grade [35]. The signal from the amorphous halos of PLA and PBAT both decreased in intensity after stretching, and reduced with a higher λ and higher T_d_ (Figure 5). The 2θ ≈ 16.2° (PBAT) and 2θ ≈ 16.5° (PLA) were difficult to distinguish from each other, and in some cases appeared to merge into one peak, but this did not compromise the crystallinity calculation as signals from both the PLA and PBAT were included.

**Figure 6:**
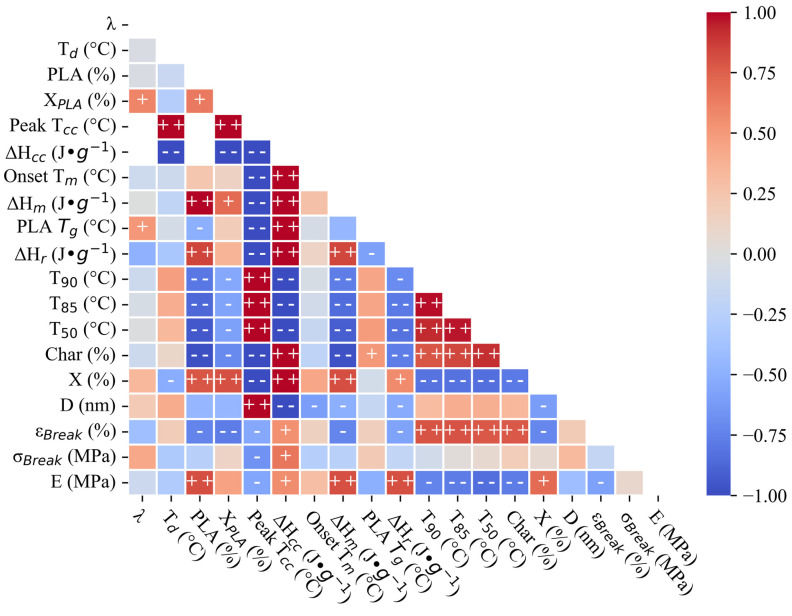
Heat map of correlation matrix for outputs where + + indicates r >0.75, + indicates 0.5 < r ≤ 0.75, − indicates −0.75 < r ≤ −0.5, and − − indicates r < −0.75. Empty boxes appear where there is insufficient data present to obtain a value for r, such as in the cases where the T_CC_ is not detectable. All the DSC parameters here are from the first heating scan. Only the DoE runs are included as the input data—namely, 1 ^D^, 2 ^D^, 3 ^D^, 4 ^D^, 5 ^D^, 6 ^D^, 7 ^D^, 8 ^D^, and 9 ^D^.

The crystallinity of PLA characterized using DSC and the overall crystallinity (PLA and PBAT) calculated using XRD (Table 3) were not equivalent but were strongly correlated (Figure 6); the XRD results were expected to be greater than those of DSC as it accounts for PBAT’s crystallinity. The crystallinity calculated using XRD was generally higher for the stretched samples than that using DSC, but it was always lower than that using DSC for the as-pressed samples. The XRD diffractograms (Figure 5) and Equation (3) were used to calculate the crystallite size. The crystallite size was negatively correlated with the crystallinity calculated using XRD (X), but not with the crystallinity calculated using DSC (X_PLA_). The blends’ crystallinity was directly correlated with the PLA concentrations, irrespective of the method used to calculate the crystallinity in the blends. The DSC X_PLA_ calculations were also highly dependent on the value used for the enthalpy of a perfect crystalline form, which varies widely in the literature [46]. The most common value which appears in the literature is 93.7 J·g^−1^ for an infinitely thick pure crystalline form of PLA [46]. Righetti et al., 2015 [46], discussed that studies have reported a wide range, from 82 to 203 J·g^−1^, and that the true reference value depends on the molecular weight, D-isomer, and crystalline (which is affected by processing) concentration of the PLA used. For example, Kalish et al. [79] extrapolated the ΔH_m_s for a 100% crystalline form and found a ${∆H}_{fusion}^{0}$(α) = 96 ± 3 J·g^−1^ and ${∆H}_{fusion}^{0}$(α’) = 57 ± 3 J·g^−1^.

### 3.4. Exploratory Statistics and Regression

A Pearson correlation matrix was depicted as a heat map (Figure 6) and used as a preliminary assessment of multicollinearity. The heatmap is a representation of the correlation coefficient between each pair of features (see Supporting Information). The PLA concentration yielded the most significant correlations out of all the input parameters. PLA correlated strongly with the ΔH_m_ (+0.99), char yield (−0.98), T_50_ (−0.94), T_85_ (−0.87), ΔH_r_ (+0.85), E (+0.81), T_90_ (−0.79), X (+0.78), ε_Break_ (−0.74), Χ_PLA_ (+0.65), and PLA *T_g_* (−0.50) (Figure 6). The ΔH_m_ correlated strongly with PLA because of the greater PLA concentrations in the blends, yielding a stronger signal. The decomposition correlations (char yield, T_50_, T_85_, and T_90_) are in agreement with those of others [58]. The PLA concentration was slightly correlated with the D (−0.45), σ_Break_ (−0.26), and T_m_ (+0.24) (Figure 6). The λ correlated strongly with fewer parameters than PLA, but more than the T_d_. The λ was strongly correlated with the Χ_PLA_ (+0.60) and PLA *T_g_* (+0.51). The λ correlated weakly with the ΔH_r_ (−0.49), σ_Break_ (+0.42), ε_Break_ (−0.39), X (+0.34), D (+0.21), E (−0.13), T_90_ (−0.12), char yield (−0.12), T_m_ (−0.11), T_85_ (−0.047), T_85_ (−0.012), and ΔH_m_ (−0.0029). The T_d_ was strongly correlated with only the X (−0.51). The T_d_ was correlated with the T_cc_ (+1) and ΔH_cc_ (−1), because very few samples exhibited cold crystallization in the first heating scan (Figure 3), yielding an artificially strong correlation. The T_d_ correlated weakly with the T_90_ (+0.47), D (+0.40), T_85_ (+0.39), T_50_ (+0.33), ΔH_r_ (−0.33), σ_Break_ (−0.31), E (−0.29), Χ_PLA_ (−0.27), ΔH_m_ (−0.20), ε_Break_ (+0.19), T_m_ (−0.12), char yield (+0.10), and PLA *T_g_* (−0.072) (Figure 6). Overall, our results suggest that the PLA concentration was more effective than the processing conditions (λ and T_d_) for controlling the film’s mechanical properties (Figure 6). Other than the processing conditions and responses, the T_85_ and char yield were strongly correlated (Figure 6): the samples exhibiting delayed decomposition also showed higher chars (Figure 2). Our results are consistent with those of other studies [58,63,80]. Any correlations with cold crystallization phenomena (T_cc_ and ΔH_cc_) were artificially perfect due to the very limited dataset of samples exhibiting cold crystallization in their first heating scan.

**Figure 7:**
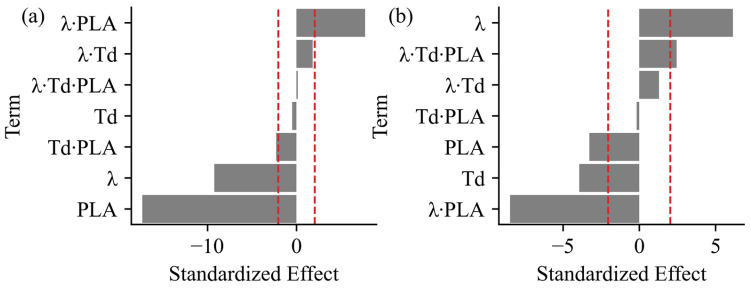
Minitab^®^ Pareto Chart of the standardized effects of terms and their interactions. Only DoE runs are included—namely, 1 ^D^, 2 ^D^, 3 ^D^, 4 ^D^, 5 ^D^, 6 ^D^, 7 ^D^, 8 ^D^, and 9 ^D^. The red dashed line at ±2.037 is the point at which the standardized effects are considered significant, assuming 95% confidence. The standardized effects are for the responses (**a**) ε_Break_ and (**b**) σ_Break_.

One limitation of correlation matrices (Figure 6) is that multicollinearity is not apparent without contextualizing the data. Therefore, further analysis using Pareto Charts (Figure 7) revealed more complex interactions between the input parameters (λ, T_d_, and PLA concentration) on the resulting properties ε_Break_ and σ_Break_. For example, the T_d_ negatively affected σ_Break,_ but had a barely significant effect on ε_Break_ (Figure 7). Furthermore, the T_d_ played less of a role in blend behavior compared to the PLA concentration (negatively effecting ε_Break_) and λ (negatively effecting ε_Break_ and positively effecting σ_Break_). These results were unexpected given that the T_d_ played a major role in the mechanical properties [17,27], but this could have been a product of our design space. Our results underscore that not only are the ε_Break_ and σ_Break_ uniquely affected by the stretching parameters, but the interaction between the input factors is complex and requires further study for optimization.

**Figure 8:**
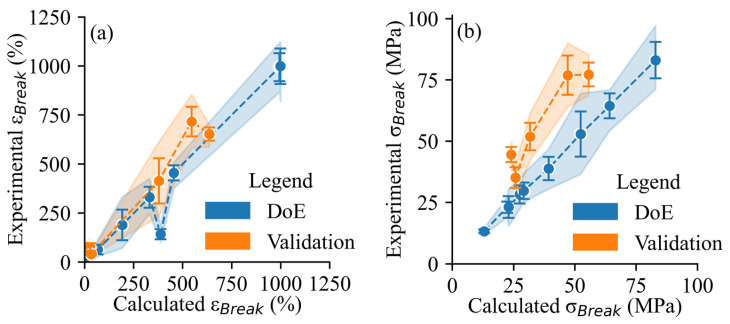
Results of regression for (**a**) ε_break_ (%), where R^2^_DOE_ = 0.96, R^2^_VALIDATION_ = 0.96; (**b**) σ_break_ (MPa), where R^2^_DOE_ = 1.0, R^2^_VALIDATION_ = 0.91. The shaded blue bands are the 95% confidence intervals for the mean prediction values. The points are the average ± standard error (n = 5) for each run. The DoE runs include 1 ^D^, 2 ^D^, 3 ^D^, 4 ^D^, 5 ^D^, 6 ^D^, 7 ^D^, 8 ^D^, and 9 ^D^. The validation runs include 19 ^V^, 20 ^V^, 21 ^V^, 22 ^V^, and 23 ^V^.

A dataset was obtained to validate the robustness of the model’s predictions (Figure 7). The predictive power of the model developed represented the ε_Break_ and σ_Break_ well, as evidenced by the high R^2^ values for both the training (DoE) and validation datasets (Figure 8).

The mechanical properties were optimized using the Minitab^®^ Result Optimizer. When the ε_Break_ and σ_Break_ were both maximized, the optimized conditions involved λ of 5 × 5, 110 °C, and 10% PLA, with a predicted ε_Break_ and σ_Break_ of 453.9% and 63.4 MPa, respectively. These conditions balanced improved strength and ductility.

## 4. Conclusions

Biodegradable polymers are critically important for reducing the plethora of microplastics in the environment and for building sustainability across agriculture, food service, packaging, biomedical engineering, and other fields. A better understanding of strategies for processing and enhancing mechanical properties of polymers and polymer blends is necessary to advance this much-needed technology. This research demonstrates that biaxial stretching is a viable technique for manipulating mechanical properties, but is less strategic than the composition based on the design space of this DoE. Specifically, the optimized conditions of 10% PLA and 90% PBAT, λ = 5 × 5, and 110 °C resulted in the greatest ε_Break_ and σ_Break_ properties. Biaxial stretching had varying effects on the thermal stability and decomposition behavior, depending on the stretching conditions and/or composition. Biaxial stretching changed the materials’ thermal signatures due to an increase in the crystalline and mesophase fractions, as evidenced by the DSC thermograms before and after erasing the materials’ thermal histories. Furthermore, XRD provided quantitative characterization of the combined PLA and PBAT crystalline phases. XRD and DSC provided evidence of PLA developing an α’ phase during stretching. PBAT also exhibited significant crystalline features, which were quantified using XRD.

## Figures and Tables

**Table 1:** Comparison of physicochemical and biodegradation properties of PLA and PBAT.

Property	PLA	PBAT	Ref.
Strength (ε_Break_)	High: up to ~90 MPa	Moderate: 14.5–61.3 MPa	[1,15,16,17,18]
Ductility (σ_Break_)	Low: 3–38%	High: 2–2500%	[1,16]
Glass transition temperature (*T_g_*)	~60 °C Brittle at ambient conditions	~−30 °C Flexible at room temperature	[1,19]
Hydrophilicity	More hydrophilic (lower water contact angle)	Less hydrophilic	[20]
Crystallinity	Higher crystallinity, thus slower biodegradation	Lower crystallinity, thus faster biodegradation	[21]
Bulk density	Denser structure, thus slower degradation	Less dense, thus more readily degradable	[21]
Environmental degradation behavior	Slower when blended with PBAT Strongly influenced by molecular weight and stereoisomer composition	Degrades faster than PLA in blends (especially in freshwater sediment)	[20,21,22]

**Table 2:** Runs 1–9 were devised by DoE software. Run 5 is a center point while the others are vertices. Runs 10–13 are control samples with only PLA. Runs 14–18 are pressed control samples (precursor to stretched samples). Runs 19–23 serve as the validation dataset.

Run ID	PLA wt%	PBAT wt%	λ	T_d_ (°C)
1 ^D^	10	90	5	110
2 ^D^	10	90	5	90
3 ^D^	10	90	2	110
4 ^D^	10	90	2	90
5 ^D^	50	50	3.5	100
6 ^D^	90	10	5	110
7 ^D^	90	10	5	90
8 ^D^	90	10	2	110
9 ^D^	90	10	2	90
10 ^C^	100	0	5	110
11 ^C^	100	0	5	90
12 ^C^	100	0	2	110
13 ^C^	100	0	2	90
14 ^C^	100	0	N/A	N/A
15 ^C^	90	10	N/A	N/A
16 ^C^	50	50	N/A	N/A
17 ^C^	10	90	N/A	N/A
18 ^C^	0	100	N/A	N/A
19 ^V^	50	50	3.5	110
20 ^V^	90	10	3	110
21 ^V^	90	10	4	110
22 ^V^	10	90	4.5	110
23 ^V^	10	90	4	110

Th superscripts ^D^, ^C^, and ^V^ represent the DoE, control, and validation datasets, respectively.

**Table 3:** Results from characterization summarized. All DSC data presented here are from first heating scan.

Run ID	DSC							TGA				XRD		Tensile		
	X_PLA_	T_cc_	ΔH_cc_	T_m_	ΔH_m_	*T_g_*	ΔH_r_	T_90_	T_85_	T_50_	Char	X	D	ε_Break_	σ_Break_	E
	%	°C	J·g^−1^	°C	J·g^−1^	°C	J·g^−1^	°C	°C	°C	%	%	nm	%	MPa	MPa
1 ^D^	22.6	*	*	160.0	3.23	70.4	0.0071	386.8	405.0	437.0	4.3	19.4	46.3	453.9	64.3	26.7
2 ^D^	21.4	*	*	157.9	3.06	66.1	0.058	362.5	382.7	421.8	4.0	22.6	60.5	330.1	83.0	69.8
3 ^D^	17.3	*	*	162.2	2.48	64.6	0.055	387.0	406.3	444.1	4.4	8.7	44.0	998.5	13.2	14.6
4 ^D^	17.7	*	*	158.2	2.53	69.1	*	380.6	394.6	425.3	5.0	12.6	39.8	908.6	12.5	8.9
5 ^D^	22.2	*	*	159.4	15.85	67.5	*	353.2	360.4	399.7	3.3	31.8	29.4	141.7	38.8	184.1
6 ^D^	23.2	*	*	160.1	29.90	64.0	0.91	351.4	357.5	377.9	1.2	36.6	53.5	35.3	28.2	299.8
7 ^D^	28.0	*	*	162.4	35.98	67.9	1.32	355.7	363.2	387.9	1.4	51.0	18.9	64.1	29.7	302.3
8 ^D^	22.9	91.3	2.4	156.6	31.81	62.6	2.39	363.7	370.3	393.8	1.6	17.4	47.7	22.2	26.1	280.2
9 ^D^	22.0	88.2	3.8	162.3	32.07	63.0	2.62	347.7	353.0	370.7	1.9	43.0	30.0	189.0	52.9	495.8
10 ^C^	20.4	93.3	1.5	161.4	30.61	63.0	0.28	345.9	354.2	378.0	0.9	24.8	85.9	20.2	26.7	253.4
11 ^C^	25.6	*	*	158.3	36.60	67.3	1.07	337.9	342.7	359.3	1.0	57.8	17.7	37.6	34.6	360.2
12 ^C^	6.0	106.2	23.4	163.0	31.99	63.5	3.56	328.6	335.1	353.6	1.7	8.3	62.3	24.3	35.5	325.6
13 ^C^	12.6	91.7	16.3	162.9	34.28	62.3	4.11	342.3	347.6	364.7	1.2	14.1	64.5	83.5	30.1	337.3
14 ^C^	2.5	107.2	29.0	156.5	32.50	51.8	1.94	350.7	356.5	374.8	1.0	1.0	90.5	19.9	49.3	330.9
15 ^C^	4.0	102.0	23.5	159.6	28.68	56.3	2.25	352.0	358.0	379.1	1.6	1.1	118.0	252.7	27.1	261.3
16 ^C^	4.4	106.0	11.7	158.8	14.85	60.5	0.98	357.8	364.5	404.4	3.8	2.5	38.9	115.9	12.1	93.6
17 ^C^	14.7	*	*	161.6	2.10	60.3	*	381.5	398.0	429.7	4.9	5.6	29.6	2449.6	27.4	27.6
18 ^C^	*	*	*	*	*	*	*	402.3	409.5	431.8	4.8	6.1	32.5	3171.3	34.1	17.7
19 ^V^	20.9	*	*	161.4	14.93	66.1	0.56	343.7	353.3	392.1	4.5	*	*	413.8	51.8	358.9
20 ^V^	23.0	*	*	159.8	29.64	65.6	1.75	319.8	335.9	369.0	1.0	*	*	76.6	44.5	491.8
21 ^V^	22.6	*	*	161.8	29.05	67.1	1.05	333.3	341.8	364.1	1.2	*	*	36.5	41.5	438.7
22 ^V^	18.4	100.8	0.3	160.7	2.91	69.1	*	368.4	384.6	417.4	6.3	*	*	716.0	77.2	76.7
23 ^V^	23.5	*	*	160.6	3.35	66.6	*	367.6	384.4	421.7	6.0	*	*	652.4	76.9	59.9

The superscripts ^D^, ^C^, and ^V^ represent the DoE, control, and validation datasets, respectively. The symbol * indicates parameters that were either unapplicable (e.g., X_PLA_ for pure PBAT), not detectable for that sample (e.g., T_CC_ for 1 ^D^), or the experiment was not performed (e.g., XRD for validation data).

## Data Availability

The original contributions presented in this study are included in the article and Supplementary Materials. Further inquiries can be directed to the corresponding author.

## Supplementary Materials

The following supporting information can be downloaded at: https://www.mdpi.com/article/10.3390/polym17192651/s1, Table S1: Design parameters for DoE; Figure S1: Engineering stress and strain curves for all runs; Table S2: Coded coefficients table for regression analysis; Figure S2: SEM images for select samples; Table S3: Analysis of variance results; Figure S3: TGA thermograms for validation dataset; Table S4 Results from pairwise Tukey’s HSD analyses for ε_Break_; Figure S4: DSC thermograms from first heating scan for validation dataset; Table S5: Results from pairwise Tukey’s HSD analyses for σ_Break_; Figure S5: DSC thermograms from second heating scan for validation dataset; Figure S6: DSC thermograms for commercial PBAT resin.

## Abbreviations

The following abbreviations are used in this manuscript:

BA	Butylene adipate
BGU	Ben-Gurion University
BT	Butylene terephthalate
D	Crystallite size
DCM	Dichloromethane
DoE	Design of experiment
DSC	Differential scanning calorimetry
DTG	Derivative thermogravimetry
MD	Machine direction
Mn	Number average molecular weight
Mw	Weight average molecular weight
NJIT	New Jersey Institute of Technology
NSF	National Science Foundation
PBAT	Poly(butylene adipate-*co*-terephthalate)
PLA	Poly(lactic acid)
SEM	Scanning electron microscopy
T90	Temperature at which 90% of mass remains (i.e., 10% mass loss)
T85	Temperature at which 85% of mass remains (i.e., 15% mass loss), taken to be the onset of decomposition
T50	Temperature at which 50% of mass remains (i.e., 50% mass loss)
TDecomp	Decomposition temperature
*Tg*	Glass transition temperature
TGA	Thermogravimetric analysis
TD	Transverse direction
ΔHcc	Enthalpy of cold crystallization
ΔH0fusion	Standard heat of fusion for infinitely thick crystalline form
ΔHm	Enthalpy of fusion
ΔHr	Enthalpic relaxation
ε_Break_	Elongation at break
λ	Draw ratio
σ_Break_	Stress at break
ΧPBAT	Fraction of crystalline PBAT
ΧPLA	Fraction of crystalline PLA
XRD	X-ray diffraction
