# Supplementary material for: Biaxial Stretching of PBAT/PLA Blends for Improved Mechanical Properties
Source: Polymers (Basel). 2025 Sep 30;17(19):2651. doi: 10.3390/polym17192651 (PMC12526632; doi:10.3390/polym17192651)
Supplement: Supplementary file 1 [file polymers-17-02651-s001.zip › polymers-3826918-supplementary.pdf]

Supporting Information

Biaxial Stretching of PBAT/PLA Blends for Improved Mechanical Properties

Nikki Rodriguez [1], Osnat Gillor [2], Murat Guvendiren [1,3], and Lisa Axe [1,*]

[1] Otto H. York Chemical and Materials Engineering Department, New Jersey Institute of Technology, Newark, NJ 07102, USA; ncr22@njit.edu (N.R.); muratg@njit.edu (M.G.)
[2] Zuckerberg Institute for Water Research, J. Blaustein Institutes for Desert Research, Ben Gurion University of the Negev, Midreshet Ben Gurion, 8499000, Israel; gilloro@bgu.ac.il
[3] Department of Biomedical Engineering, New Jersey Institute of Technology, Newark, NJ 07102, USA
* Correspondence: axe@njit.edu

Design of Experiment

The design parameters for the Design of Experiment (DoE) are as follows in Table S1.

Table S1: Design parameters for DOE.

Factor	Lower Limit	Upper Limit
λ	2	5
T_d (°C)	90	110
PLA wt%	10	90

The Pearson correlation coefficient (r) was used during exploratory statistics to assess linear correlations between pairs of parameters, as given by:

$$r = \frac{\sum_{i=1}^{n}(x_i - \bar{x})(y_i - \bar{y})}{\sqrt{\sum_{i=1}^{n}(x_i - \bar{x})^2 \sum_{i=1}^{n}(y_i - \bar{y})^2}} \quad \text{(S1)}$$

where n is the sample size, x_i and y_i are the sample points indexed using i, and $\bar{x}$ and $\bar{y}$ are the sample means. Regression was performed between mechanical responses (ε_{Break} (%), σ_{Break} (MPa)) and the predictors (λ, T_d, PLA concentration). P-values for regression were obtained assuming a null hypothesis where all coefficients were zero and an alternate hypothesis where coefficients were nonzero. For example, for a regression equation with dependent variable y, independent variables x_1, $x_2 \ldots x_n$ and coefficients β_0, β_1, $\beta_2, \ldots, \beta_n$ represented by:

$$y = \beta_0 + \beta_1 x_1 + \beta_2 x_2 + \cdots + \beta_n x_n \quad \text{(S2)}$$

the null hypothesis would be $\beta_1 = \beta_2 = \ldots = \beta_n = 0$ while the alternate hypothesis is $\beta_i \neq 0$. For brevity, λ is denoted x_1, T_d as x_2, and PLA concentration as x_3 in all representations. Additionally, capital letters (A-H) are reserved for representing fitted coefficients while lowercase letters (x, y) are used for (in)dependent variables. Special cubic model regression was

performed using Equation S3:

$$y = A(x_1) + B(x_2) + C(x_3) + D(x_1x_2) + E(x_1x_3) + F(x_2x_3) + G(x_1x_2x_3) + H \quad \text{(S3)}$$

where x_1, x_2, and x_3 are represented by λ, T_d, PLA, respectively. Regression analyses on ε_{Break} and σ_{Break} with independent variables (λ, T_d, PLA concentration) was performed using polynomial regression. Uncoded coefficients from Table S2 were used to calculate/predict parameters (ε_{Break} and σ_{Break}) when plugged into Equation S3.

Table S2. Coded Coefficients table using Minitab®. Parameters include effect, uncoded coefficient (UC), coded coefficient (CC), standard error of coded coefficient (SE CC), 95% confidence interval (CI), t-value, p-value, and Variance Inflation Factor (VIF).

Parameter	Term	Effect	UC	CC	SE CC	95% CI	t-value	p-value	VIF
ε_{Break} (%)	H		1811	385.5	17.8	349.2, 421.9	21.6	0	
	A	-329.3	-418	-164.6	17.8	-201.0, -128.3	-9.23	0	1
	B	-17.5	-2.55	-8.7	17.8	-45.1, 27.6	-0.49	0.628	1
	C	-617.1	-3.9	-308.6	17.8	-344.9, -272.2	-17.29	0	1
	D	65	1.94	32.5	17.8	-3.9, 68.8	1.82	0.078	1
	E	274.8	1.84	137.4	17.8	101.0, 173.8	7.7	0	1
	F	-81.7	-0.118	-40.9	17.8	-77.2, -4.5	-2.29	0.029	1
	G	5.4	0.0045	2.7	17.8	-33.6, 39.1	0.15	0.881	1
	Center Point		-243.8	-243.8	53.5	-352.9, -134.7	-4.55	0	1
σ_{Break} (MPa)	H		-34.5	39.69	1.9	35.82, 43.56	20.88	0	
	A	23.3	43.6	11.65	1.9	7.78, 15.52	6.13	0	1
	B	-14.98	0.077	-7.49	1.9	-11.36, -3.62	-3.94	0	1
	C	-12.45	3.58	-6.23	1.9	-10.10, -2.36	-3.28	0.003	1
	D	4.9	-0.224	2.45	1.9	-1.42, 6.32	1.29	0.207	1
	E	-32.25	-1.043	-16.12	1.9	-20.00, -12.25	-8.48	0	1
	F	-0.69	-0.0280	-0.34	1.9	-4.22, 3.53	-0.18	0.858	1
	G	9.29	0.00774	4.64	1.9	0.77, 8.52	2.44	0.02	1
	Center Point		-0.90	-0.9	5.7	-12.51, 10.72	-0.16	0.876	1

Table S3. Analysis of Variance output using Minitab®.

Parameter	Source	DF	Seq SS	Contribution	Adj SS	Adj MS	F-Value	P-Value
εBreak (%)	Model	12	6118646	93.75%	6118646	509887	40.02	0
	Linear	4	93961	1.44%	93961	23490	1.84	0.145
	A	3	4896102	75.02%	4896102	1632034	128.1	0
	B	1	1084320	16.61%	1084320	1084320	85.11	0
	C	1	3055	0.05%	3055	3055	0.24	0.628
	2-Way Interactions	1	3808727	58.36%	3808727	3808727	298.94	0
	D	3	864135	13.24%	864135	288045	22.61	0
	E	1	42186	0.65%	42186	42186	3.31	0.078
	F	1	755122	11.57%	755122	755122	59.27	0
	3-Way Interactions	1	66827	1.02%	66827	66827	5.25	0.029

	G	1	293	0.00%	293	293	0.02	0.881
	Curvature	1	293	0.00%	293	293	0.02	0.881
	Error	1	264156	4.05%	264156	264156	20.73	0
	Total	32	407701	6.25%	407701	12741		
σBreak (MPa)	Model	12	20892.5	81.88%	20892.5	1741	12.05	0
	Linear	4	160	0.63%	160	40	0.28	0.891
	A	3	9222	36.14%	9222	3074	21.27	0
	B	1	5428	21.27%	5428	5428	37.56	0
	C	1	2242.9	8.79%	2242.9	2242.9	15.52	0
	2-Way Interactions	1	1551.1	6.08%	1551.1	1551.1	10.73	0.003
	D	3	10644.1	41.71%	10644.1	3548	24.55	0
	E	1	240	0.94%	240	240	1.66	0.207
	F	1	10399.4	40.76%	10399.4	10399.4	71.97	0
	3-Way Interactions	1	4.7	0.02%	4.7	4.7	0.03	0.858
	G	1	862.9	3.38%	862.9	862.9	5.97	0.02
	Curvature	1	862.9	3.38%	862.9	862.9	5.97	0.02
	Error	1	3.6	0.01%	3.6	3.6	0.02	0.876
	Total	32	4624.2	18.12%	4624.2	144.5		

Mechanical testing

Engineering stress-strain curves were used to calculate ε_{Break} and σ_{Break}. Percent ε_{Break} was calculated using L_{Break}, the extension or gage length of the sample at break (mm), and L_0, the initial gage length (not to be confused with initial grip separation) of the sample at the start of the experiment (mm) [1] (Equation S4).

$$\varepsilon_{Break} = \frac{L_{Break}}{L_0} \cdot 100 \tag{S4}$$

σ_{Break} was calculated from F_{Break}, the force when the sample has broken, and A, the cross-sectional area of the sample's gage portion [1] (Equation S5).

$$\sigma_{Break} = \frac{F_{Break}}{A} \tag{S5}$$

The full stress-strain curves for each sample are also provided (Figure S1).

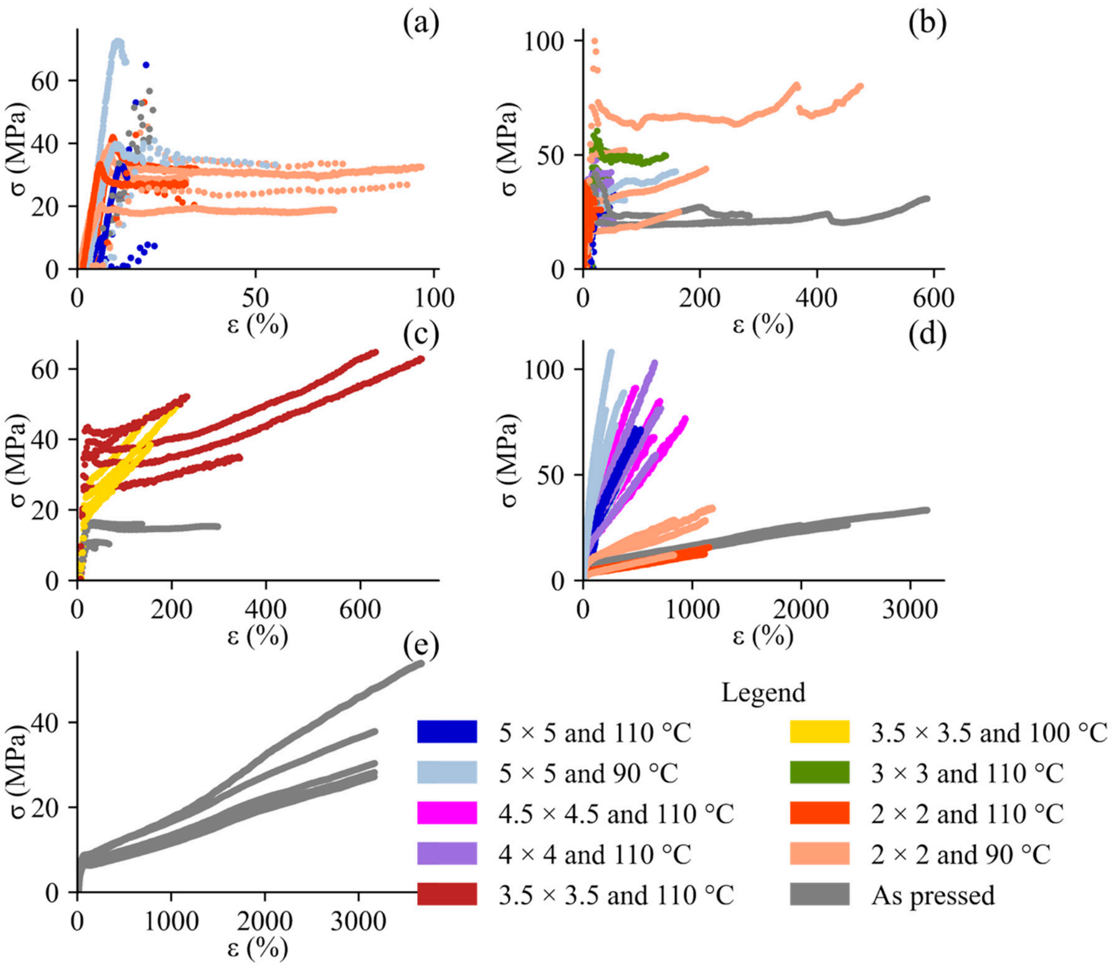

Figure S1. Engineering stress and strain curves (n=5) for all runs, organized by composition as in: (a) pure PLA, (b) 90%/10% PLA/PBAT, (c) 50%/50% PLA/PBAT, (d) 10%/90% PLA/PBAT, and (e) pure PBAT.

The Tukey Honestly Significant Difference (HSD) analyses can be found for ε_{Break} (Table S4) and σ_{Break} (Table S5) below.

Table S4. Results from Tukey HSD analyses between each pair of runs for ε_{Break}.

Group 1 (Run #)	Group 2 (Run #)	Mean diff	p-adj	lower	upper	reject
7	17	2385.458	0	2008.752	2762.164	TRUE
15	18	2918.554	0	2420.219	3416.888	TRUE
5	23	510.6516	0.0006	133.9461	887.3571	TRUE
5	22	574.2638	0.0001	197.5582	950.9693	TRUE
13	18	3087.823	0	2492.199	3683.447	TRUE
5	18	3029.567	0	2531.233	3527.902	TRUE
5	17	2307.862	0	1931.157	2684.568	TRUE
13	22	632.5198	0.0019	134.1852	1130.854	TRUE
13	23	568.9076	0.0098	70.5731	1067.242	TRUE
14	17	2429.636	0	2052.93	2806.341	TRUE

14	18	3151.34	0	2653.006	3649.675	TRUE
14	19	393.8552	0.0304	17.1496	770.5607	TRUE
14	22	696.0369	0	319.3314	1072.742	TRUE
14	23	632.4248	0	255.7192	1009.13	TRUE
4	20	-831.96	0	-1330.29	-333.625	TRUE
15	17	2196.849	0	1820.143	2573.554	TRUE
4	18	2262.689	0	1667.066	2858.313	TRUE
4	17	1540.985	0	1042.65	2039.319	TRUE
4	16	-792.712	0	-1291.05	-294.377	TRUE
4	15	-655.864	0.001	-1154.2	-157.53	TRUE
4	14	-888.651	0	-1386.99	-390.316	TRUE
4	13	-825.134	0.0004	-1420.76	-229.51	TRUE
4	12	-884.306	0	-1428.03	-340.578	TRUE
4	11	-870.955	0	-1414.68	-327.227	TRUE
4	10	-888.414	0	-1432.14	-344.686	TRUE
4	9	-719.619	0.0002	-1217.95	-221.285	TRUE
4	8	-886.439	0	-1430.17	-342.711	TRUE
4	7	-844.473	0	-1342.81	-346.139	TRUE
4	6	-873.341	0	-1371.68	-375.006	TRUE
13	17	2366.118	0	1867.784	2864.453	TRUE
12	23	628.0798	0.0002	193.0977	1063.062	TRUE
12	22	691.6919	0	256.7098	1126.674	TRUE
7	23	588.2472	0	211.5417	964.9527	TRUE
9	18	2982.308	0	2483.974	3480.643	TRUE
9	17	2260.604	0	1883.898	2637.309	TRUE
9	22	527.0051	0.0003	150.2995	903.7106	TRUE
9	23	463.3929	0.0032	86.6874	840.0984	TRUE
8	23	630.2128	0.0002	195.2307	1065.195	TRUE
8	22	693.8249	0	258.8428	1128.807	TRUE
10	17	2429.399	0	1994.416	2864.381	TRUE
10	18	3151.103	0	2607.376	3694.831	TRUE
8	18	3149.128	0	2605.401	3692.856	TRUE
8	17	2427.424	0	1992.441	2862.406	TRUE
10	22	695.7999	0	260.8178	1130.782	TRUE
10	23	632.1878	0.0001	197.2057	1067.17	TRUE
7	22	651.8594	0	275.1538	1028.565	TRUE
12	18	3146.995	0	2603.268	3690.723	TRUE
11	17	2411.939	0	1976.957	2846.921	TRUE
7	18	3107.163	0	2608.828	3605.497	TRUE
1	3	544.5432	0.0002	167.8377	921.2487	TRUE
11	18	3133.644	0	2589.916	3677.372	TRUE
11	22	678.3407	0	243.3586	1113.323	TRUE
11	23	614.7285	0.0003	179.7464	1049.711	TRUE

6	23	617.1142	0	240.4087	993.8198	TRUE
6	22	680.7264	0	304.0209	1057.432	TRUE
12	17	2425.291	0	1990.308	2860.273	TRUE
6	19	378.5446	0.0475	1.8391	755.2502	TRUE
6	18	3136.03	0	2637.695	3634.364	TRUE
6	17	2414.325	0	2037.62	2791.031	TRUE
4	5	-766.878	0	-1265.21	-268.543	TRUE
4	21	-872.061	0.0001	-1467.68	-276.437	TRUE
17	19	-2035.78	0	-2412.49	-1659.07	TRUE
17	21	-2413.05	0	-2911.38	-1914.71	TRUE
2	18	2841.148	0	2342.814	3339.483	TRUE
2	17	2119.443	0	1742.738	2496.149	TRUE
16	18	3055.401	0	2557.066	3553.735	TRUE
20	22	639.3454	0	262.6399	1016.051	TRUE
16	22	600.0975	0	223.392	976.803	TRUE
16	23	536.4854	0.0002	159.7798	913.1909	TRUE
17	18	721.7047	0.0002	223.3702	1220.039	TRUE
1	14	-434.004	0.0086	-810.709	-57.2983	TRUE
3	21	-961.957	0	-1460.29	-463.622	TRUE
17	20	-2372.94	0	-2749.65	-1996.24	TRUE
17	22	-1733.6	0	-2110.3	-1356.89	TRUE
15	23	399.6381	0.0256	22.9326	776.3436	TRUE
17	23	-1797.21	0	-2173.92	-1420.51	TRUE
2	4	578.4588	0.0077	80.1242	1076.793	TRUE
2	3	668.3549	0	291.6493	1045.06	TRUE
18	19	-2757.49	0	-3255.82	-2259.15	TRUE
18	20	-3094.65	0	-3592.98	-2596.31	TRUE
18	23	-2518.92	0	-3017.25	-2020.58	TRUE
1	17	1995.632	0	1618.926	2372.337	TRUE
18	21	-3134.75	0	-3730.37	-2539.13	TRUE
1	20	-377.312	0.0492	-754.018	-0.6069	TRUE
18	22	-2455.3	0	-2953.64	-1956.97	TRUE
16	17	2333.696	0	1956.991	2710.402	TRUE
20	23	575.7333	0	199.0278	952.4388	TRUE
15	22	463.2503	0.0032	86.5447	839.9558	TRUE
3	13	-915.03	0	-1413.36	-416.695	TRUE
3	7	-934.37	0	-1311.08	-557.664	TRUE
3	8	-976.335	0	-1411.32	-541.353	TRUE
3	9	-809.515	0	-1186.22	-432.81	TRUE
3	10	-978.31	0	-1413.29	-543.328	TRUE
3	11	-960.851	0	-1395.83	-525.869	TRUE
3	12	-974.202	0	-1409.18	-539.22	TRUE
3	14	-978.547	0	-1355.25	-601.842	TRUE

21	23	615.8344	0.003	117.4998	1114.169	TRUE
3	15	-745.76	0	-1122.47	-369.055	TRUE
3	16	-882.608	0	-1259.31	-505.902	TRUE
3	17	1451.088	0	1074.383	1827.794	TRUE
3	18	2172.793	0	1674.459	2671.128	TRUE
3	19	-584.692	0	-961.397	-207.986	TRUE
3	20	-921.856	0	-1298.56	-545.15	TRUE
3	5	-856.774	0	-1233.48	-480.068	TRUE
3	6	-963.237	0	-1339.94	-586.531	TRUE
1	18	2717.336	0	2219.002	3215.671	TRUE
2	22	385.8447	0.0385	9.1392	762.5502	TRUE
21	22	679.4465	0.0005	181.112	1177.781	TRUE
1	7	-389.826	0.0343	-766.532	-13.1208	TRUE
1	6	-418.693	0.0141	-795.399	-41.9878	TRUE
11	13	45.8209	1	-497.907	589.5485	FALSE
10	14	-0.237	1	-435.219	434.7451	FALSE
10	12	4.108	1	-482.217	490.4327	FALSE
11	15	215.0904	0.9633	-219.892	650.0725	FALSE
10	15	232.5496	0.9238	-202.432	667.5317	FALSE
10	11	17.4592	1	-468.866	503.784	FALSE
11	16	78.2432	1	-356.739	513.2252	FALSE
11	14	-17.6962	1	-452.678	417.2858	FALSE
10	19	393.6181	0.1284	-41.3639	828.6002	FALSE
10	20	56.4544	1	-378.528	491.4365	FALSE
10	16	95.7024	1	-339.28	530.6845	FALSE
19	20	-337.164	0.1404	-713.869	39.5418	FALSE
20	21	-40.1011	1	-538.436	458.2335	FALSE
19	23	238.5696	0.7358	-138.136	615.2751	FALSE
19	22	302.1818	0.2989	-74.5238	678.8873	FALSE
19	21	-377.265	0.4051	-875.599	121.0698	FALSE
10	13	63.2801	1	-480.448	607.0077	FALSE
10	21	16.3534	1	-527.374	560.081	FALSE
11	12	-13.3512	1	-499.676	472.9735	FALSE
15	19	161.0685	0.9927	-215.637	537.774	FALSE
11	19	376.1589	0.1829	-58.8232	811.141	FALSE
11	20	38.9952	1	-395.987	473.9773	FALSE
9	20	-112.34	1	-489.046	264.3651	FALSE
15	16	-136.847	0.9992	-513.553	239.8583	FALSE
15	20	-176.095	0.9796	-552.801	200.6103	FALSE
15	21	-216.196	0.9913	-714.531	282.1383	FALSE
14	21	16.5904	1	-481.744	514.9249	FALSE
14	20	56.6914	1	-320.014	433.397	FALSE
14	16	95.9394	1	-280.766	472.6449	FALSE

14	15	232.7866	0.7728	-143.919	609.4922	FALSE
13	21	-46.9267	1	-642.551	548.697	FALSE
13	20	-6.8257	1	-505.16	491.5089	FALSE
13	19	330.338	0.6592	-167.997	828.6726	FALSE
16	19	297.9157	0.3239	-78.7898	674.6213	FALSE
16	20	-39.248	1	-415.954	337.4576	FALSE
16	21	-79.349	1	-577.684	418.9855	FALSE
13	16	32.4223	1	-465.912	530.7568	FALSE
13	15	169.2695	0.9997	-329.065	667.6041	FALSE
13	14	-63.5171	1	-561.852	434.8174	FALSE
12	21	12.2454	1	-531.482	555.973	FALSE
12	20	52.3464	1	-382.636	487.3285	FALSE
12	19	389.5101	0.1399	-45.4719	824.4922	FALSE
12	16	91.5944	1	-343.388	526.5765	FALSE
12	15	228.4416	0.9349	-206.54	663.4237	FALSE
12	14	-4.345	1	-439.327	430.6371	FALSE
12	13	59.1721	1	-484.556	602.8997	FALSE
11	21	-1.1059	1	-544.834	542.6217	FALSE
9	21	-152.442	0.9999	-650.776	345.8931	FALSE
1	2	-123.812	0.9998	-500.517	252.8939	FALSE
9	19	224.8233	0.8196	-151.882	601.5288	FALSE
4	19	-494.796	0.0539	-993.13	3.5387	FALSE
2	15	-77.4055	1	-454.111	299.3	FALSE
2	16	-214.253	0.8732	-590.958	162.4527	FALSE
2	19	83.663	1	-293.043	460.3685	FALSE
2	20	-253.501	0.632	-630.206	123.2048	FALSE
2	21	-293.602	0.8352	-791.936	204.7327	FALSE
2	23	322.2326	0.1978	-54.473	698.9381	FALSE
3	4	-89.8961	1	-588.231	408.4385	FALSE
3	22	-282.51	0.4233	-659.216	94.1954	FALSE
3	23	-346.122	0.1128	-722.828	30.5832	FALSE
4	22	-192.614	0.998	-690.949	305.7205	FALSE
2	13	-246.675	0.9629	-745.01	251.6595	FALSE
4	23	-256.226	0.9465	-754.561	242.1083	FALSE
5	6	-106.463	1	-483.168	270.2429	FALSE
5	7	-77.5956	1	-454.301	299.1099	FALSE
5	8	-119.561	1	-554.543	315.4209	FALSE
5	9	47.2587	1	-329.447	423.9642	FALSE
5	10	-121.536	1	-556.518	313.4459	FALSE
5	11	-104.077	1	-539.059	330.9052	FALSE
5	12	-117.428	1	-552.41	317.5539	FALSE
5	13	-58.256	1	-556.591	440.0785	FALSE
2	14	-310.192	0.2552	-686.898	66.5133	FALSE

2	12	-305.847	0.5491	-740.829	129.1349	FALSE
9	16	-73.0924	1	-449.798	303.6131	FALSE
1	16	-338.064	0.1374	-714.77	38.6411	FALSE
1	4	454.6472	0.1199	-43.6874	952.9817	FALSE
1	5	-312.231	0.2447	-688.936	64.4748	FALSE
1	8	-431.792	0.054	-866.774	3.1902	FALSE
1	9	-264.972	0.5484	-641.678	111.7335	FALSE
1	10	-433.767	0.0515	-868.749	1.2152	FALSE
1	11	-416.308	0.0779	-851.29	18.6745	FALSE
1	12	-429.659	0.0569	-864.641	5.3232	FALSE
1	13	-370.487	0.4402	-868.821	127.8478	FALSE
1	15	-201.217	0.9244	-577.923	175.4883	FALSE
1	19	-40.1487	1	-416.854	336.5568	FALSE
2	11	-292.496	0.6334	-727.478	142.4861	FALSE
1	21	-417.414	0.2284	-915.748	80.9211	FALSE
1	22	262.0331	0.5699	-114.673	638.7386	FALSE
1	23	198.4209	0.9332	-178.285	575.1264	FALSE
2	5	-188.419	0.9589	-565.125	188.2865	FALSE
2	6	-294.882	0.3424	-671.587	81.8238	FALSE
2	7	-266.015	0.5407	-642.72	110.6909	FALSE
2	8	-307.98	0.5356	-742.962	127.0019	FALSE
2	9	-141.16	0.9987	-517.866	235.5452	FALSE
2	10	-309.955	0.5232	-744.937	125.0269	FALSE
5	14	-121.773	0.9999	-498.479	254.9324	FALSE
5	15	111.0135	1	-265.692	487.719	FALSE
5	16	-25.8337	1	-402.539	350.8718	FALSE
8	15	230.5746	0.9293	-204.407	665.5567	FALSE
7	19	349.6776	0.1032	-27.0279	726.3831	FALSE
7	20	12.5139	1	-364.192	389.2194	FALSE
7	21	-27.5872	1	-525.922	470.7474	FALSE
8	9	166.8198	0.9982	-268.162	601.8019	FALSE
8	10	-1.975	1	-488.3	484.3497	FALSE
8	11	15.4842	1	-470.841	501.809	FALSE
8	12	2.133	1	-484.192	488.4577	FALSE
8	13	61.3051	1	-482.423	605.0327	FALSE
8	14	-2.212	1	-437.194	432.7701	FALSE
8	16	93.7274	1	-341.255	528.7095	FALSE
5	19	272.082	0.4967	-104.624	648.7875	FALSE
8	19	391.6432	0.1338	-43.3389	826.6252	FALSE
8	20	54.4794	1	-380.503	489.4615	FALSE
8	21	14.3784	1	-529.349	558.106	FALSE
9	10	-168.795	0.9979	-603.777	266.1872	FALSE
9	11	-151.336	0.9996	-586.318	283.6465	FALSE

9	12	-164.687	0.9985	-599.669	270.2952	FALSE
9	13	-105.515	1	-603.849	392.8198	FALSE
9	14	-169.032	0.9871	-545.737	207.6737	FALSE
9	15	63.7548	1	-312.951	440.4603	FALSE
7	16	51.7619	1	-324.944	428.4674	FALSE
7	15	188.6091	0.9585	-188.096	565.3146	FALSE
7	14	-44.1776	1	-420.883	332.528	FALSE
7	13	19.3396	1	-478.995	517.6741	FALSE
5	20	-65.0817	1	-441.787	311.6238	FALSE
5	21	-105.183	1	-603.517	393.1518	FALSE
6	7	28.8671	1	-347.839	405.5726	FALSE
6	8	-13.0985	1	-448.081	421.8836	FALSE
6	9	153.7213	0.9959	-222.984	530.4269	FALSE
6	10	-15.0735	1	-450.056	419.9086	FALSE
6	11	2.3858	1	-432.596	437.3678	FALSE
6	12	-10.9655	1	-445.948	424.0166	FALSE
6	13	48.2066	1	-450.128	546.5412	FALSE
6	14	-15.3105	1	-392.016	361.395	FALSE
6	15	217.4761	0.858	-159.229	594.1817	FALSE
6	16	80.6289	1	-296.077	457.3344	FALSE
6	20	41.381	1	-335.325	418.0865	FALSE
6	21	1.2799	1	-497.055	499.6144	FALSE
7	8	-41.9656	1	-476.948	393.0165	FALSE
7	9	124.8543	0.9998	-251.851	501.5598	FALSE
7	10	-43.9406	1	-478.923	391.0415	FALSE
7	11	-26.4813	1	-461.463	408.5008	FALSE
7	12	-39.8326	1	-474.815	395.1495	FALSE
22	23	-63.6122	1	-440.318	313.0934	FALSE

Table S5. Results from Tukey HSD analyses between each pair of runs for σ_{Break}.

Group 1 (Run #)	Group 2 (Run #)	Mean diff	p-adj	lower	upper	reject
16	22	65.0341	0	36.7487	93.3196	TRUE
3	9	39.6691	0.0003	11.3836	67.9545	TRUE
7	22	47.4497	0	19.1643	75.7351	TRUE
7	23	47.1681	0	18.8827	75.4536	TRUE
16	19	39.7021	0.0003	11.4167	67.9876	TRUE
16	20	32.3889	0.0094	4.1035	60.6744	TRUE
3	14	36.0706	0.0017	7.7852	64.3561	TRUE
16	23	64.7526	0	36.4671	93.038	TRUE
12	23	41.3658	0.002	8.7045	74.027	TRUE
2	16	-70.8883	0	-99.1737	-42.6028	TRUE
17	22	49.733	0	21.4476	78.0184	TRUE

17	23	49.4514	0	21.166	77.7369	TRUE
12	22	41.6473	0.0017	8.9861	74.3086	TRUE
2	21	-41.5236	0.0144	-78.9417	-4.1055	TRUE
2	19	-31.1861	0.0157	-59.4716	-2.9007	TRUE
2	18	-48.9319	0.0011	-86.35	-11.5138	TRUE
3	19	38.6488	0.0005	10.3634	66.9343	TRUE
3	20	31.3356	0.0148	3.0502	59.6211	TRUE
15	23	49.7668	0	21.4813	78.0522	TRUE
3	22	63.9808	0	35.6954	92.2663	TRUE
3	23	63.6992	0	35.4138	91.9847	TRUE
15	22	50.0484	0	21.7629	78.3338	TRUE
6	22	48.9253	0	20.6399	77.2108	TRUE
6	23	48.6437	0	20.3583	76.9292	TRUE
4	9	40.3411	0.021	2.923	77.7593	TRUE
13	22	47.0531	0.0022	9.635	84.4712	TRUE
1	3	-51.1561	0	-79.4416	-22.8707	TRUE
13	23	46.7715	0.0024	9.3534	84.1897	TRUE
4	23	64.3713	0	26.9532	101.7894	TRUE
4	22	64.6529	0	27.2348	102.071	TRUE
10	22	50.517	0	17.8558	83.1782	TRUE
10	23	50.2354	0	17.5742	82.8966	TRUE
14	16	-37.124	0.0011	-65.4094	-8.8385	TRUE
2	17	-55.5871	0	-83.8726	-27.3017	TRUE
2	20	-38.4993	0.0005	-66.7848	-10.2139	TRUE
2	15	-55.9025	0	-84.1879	-27.6171	TRUE
11	22	42.6102	0.0012	9.949	75.2715	TRUE
18	23	42.7962	0.0096	5.3781	80.2143	TRUE
11	23	42.3287	0.0013	9.6674	74.9899	TRUE
5	22	38.3827	0.0006	10.0972	66.6681	TRUE
9	16	-40.7224	0.0002	-69.0079	-12.437	TRUE
1	17	-36.9083	0.0012	-65.1937	-8.6228	TRUE
1	16	-52.2094	0	-80.4949	-23.924	TRUE
1	15	-37.2237	0.001	-65.5091	-8.9382	TRUE
5	23	38.1011	0.0007	9.8157	66.3865	TRUE
20	22	32.6452	0.0084	4.3598	60.9307	TRUE
20	23	32.3636	0.0095	4.0782	60.6491	TRUE
1	10	-37.6923	0.0084	-70.3535	-5.031	TRUE
8	22	51.0369	0	18.3757	83.6981	TRUE
1	8	-38.2122	0.0069	-70.8734	-5.551	TRUE
1	7	-34.625	0.0034	-62.9104	-6.3395	TRUE
1	6	-36.1006	0.0017	-64.3861	-7.8152	TRUE
8	23	50.7553	0	18.0941	83.4165	TRUE
1	4	-51.8282	0.0004	-89.2463	-14.41	TRUE

2	14	-33.7643	0.0051	-62.0498	-5.4789	TRUE
18	22	43.0778	0.0087	5.6596	80.4959	TRUE
2	3	-69.835	0	-98.1204	-41.5495	TRUE
2	4	-70.507	0	-107.925	-33.0889	TRUE
2	13	-52.9072	0.0003	-90.3254	-15.4891	TRUE
2	12	-47.5015	0.0001	-80.1627	-14.8402	TRUE
2	11	-48.4644	0.0001	-81.1256	-15.8032	TRUE
2	10	-56.3711	0	-89.0323	-23.7099	TRUE
2	9	-30.1659	0.024	-58.4513	-1.8804	TRUE
2	8	-56.891	0	-89.5522	-24.2298	TRUE
2	7	-53.3038	0	-81.5893	-25.0184	TRUE
2	6	-54.7795	0	-83.0649	-26.494	TRUE
2	5	-44.2368	0	-72.5223	-15.9514	TRUE
4	19	39.3209	0.0286	1.9027	76.739	TRUE
11	20	9.965	0.9999	-22.6962	42.6263	FALSE
12	13	-5.4058	1	-46.2323	35.4207	FALSE
11	15	-7.4381	1	-40.0993	25.2231	FALSE
11	19	17.2782	0.9306	-15.383	49.9395	FALSE
11	18	-0.4675	1	-41.294	40.359	FALSE
11	14	14.7001	0.9866	-17.9612	47.3613	FALSE
11	21	6.9408	1	-33.8857	47.7673	FALSE
11	13	-4.4429	1	-45.2694	36.3837	FALSE
11	17	-7.1228	1	-39.784	25.5385	FALSE
11	16	-22.4239	0.5947	-55.0851	10.2373	FALSE
1	2	18.6788	0.6659	-9.6066	46.9643	FALSE
11	12	0.9629	1	-35.5534	37.4793	FALSE
10	11	7.9067	1	-28.6096	44.4231	FALSE
9	14	-3.5984	1	-31.8839	24.687	FALSE
9	15	-25.7366	0.1226	-54.0221	2.5488	FALSE
9	17	-25.4213	0.1358	-53.7067	2.8642	FALSE
9	18	-18.766	0.9578	-56.1842	18.6521	FALSE
9	19	-1.0203	1	-29.3057	27.2652	FALSE
9	20	-8.3335	1	-36.6189	19.952	FALSE
9	21	-11.3577	0.9999	-48.7759	26.0604	FALSE
9	22	24.3117	0.1911	-3.9737	52.5972	FALSE
9	23	24.0302	0.2075	-4.2553	52.3156	FALSE
10	12	8.8696	1	-27.6467	45.386	FALSE
10	21	14.8475	0.9991	-25.979	55.674	FALSE
12	15	-8.401	1	-41.0623	24.2602	FALSE
10	13	3.4639	1	-37.3627	44.2904	FALSE
10	14	22.6068	0.5793	-10.0544	55.268	FALSE
10	15	0.4686	1	-32.1926	33.1298	FALSE
10	16	-14.5172	0.9884	-47.1784	18.1441	FALSE

10	17	0.784	1	-31.8772	33.4452	FALSE
10	18	7.4392	1	-33.3873	48.2657	FALSE
10	19	25.185	0.3703	-7.4762	57.8462	FALSE
10	20	17.8718	0.9071	-14.7895	50.533	FALSE
12	14	13.7372	0.994	-18.9241	46.3984	FALSE
14	20	-4.735	1	-33.0205	23.5504	FALSE
12	16	-23.3868	0.5137	-56.048	9.2744	FALSE
17	21	14.0635	0.9986	-23.3546	51.4817	FALSE
15	19	24.7164	0.1693	-3.5691	53.0018	FALSE
15	20	17.4031	0.779	-10.8823	45.6886	FALSE
15	21	14.3789	0.9982	-23.0392	51.797	FALSE
16	17	15.3011	0.9156	-12.9843	43.5866	FALSE
16	18	21.9564	0.84	-15.4618	59.3745	FALSE
16	21	29.3647	0.3378	-8.0535	66.7828	FALSE
17	18	6.6552	1	-30.7629	44.0734	FALSE
17	19	24.401	0.1861	-3.8845	52.6864	FALSE
17	20	17.0878	0.804	-11.1977	45.3732	FALSE
18	19	17.7458	0.9761	-19.6724	55.1639	FALSE
12	17	-8.0857	1	-40.7469	24.5756	FALSE
18	20	10.4326	1	-26.9856	47.8507	FALSE
18	21	7.4083	1	-37.3149	52.1315	FALSE
19	20	-7.3132	1	-35.5987	20.9722	FALSE
19	21	-10.3375	1	-47.7556	27.0807	FALSE
19	22	25.332	0.1397	-2.9534	53.6175	FALSE
19	23	25.0504	0.1527	-3.235	53.3359	FALSE
20	21	-3.0243	1	-40.4424	34.3939	FALSE
21	22	35.6695	0.0809	-1.7487	73.0876	FALSE
21	23	35.3879	0.0871	-2.0302	72.806	FALSE
15	18	6.9706	1	-30.4475	44.3887	FALSE
15	17	0.3154	1	-27.9701	28.6008	FALSE
15	16	-14.9858	0.9296	-43.2712	13.2997	FALSE
14	23	27.6286	0.0637	-0.6568	55.914	FALSE
12	18	-1.4304	1	-42.257	39.3961	FALSE
12	19	16.3153	0.9594	-16.3459	48.9765	FALSE
12	20	9.0021	1	-23.6591	41.6633	FALSE
12	21	5.9779	1	-34.8487	46.8044	FALSE
13	14	19.1429	0.9489	-18.2752	56.5611	FALSE
13	15	-2.9952	1	-40.4134	34.4229	FALSE
13	16	-17.981	0.9726	-55.3992	19.4371	FALSE
13	17	-2.6799	1	-40.098	34.7382	FALSE
13	18	3.9753	1	-40.7479	48.6986	FALSE
13	19	21.7211	0.852	-15.697	59.1392	FALSE
13	20	14.4079	0.9981	-23.0102	51.826	FALSE

13	21	11.3836	1	-33.3396	56.1069	FALSE
14	15	-22.1382	0.3427	-50.4236	6.1473	FALSE
14	17	-21.8228	0.3693	-50.1083	6.4626	FALSE
14	18	-15.1676	0.9963	-52.5857	22.2505	FALSE
14	19	2.5782	1	-25.7073	30.8636	FALSE
9	12	-17.3356	0.9285	-49.9968	15.3256	FALSE
14	21	-7.7593	1	-45.1774	29.6588	FALSE
14	22	27.9102	0.0575	-0.3753	56.1956	FALSE
9	13	-22.7414	0.796	-60.1595	14.6768	FALSE
7	17	-2.2833	1	-30.5687	26.0021	FALSE
9	11	-18.2985	0.8873	-50.9597	14.3627	FALSE
4	14	36.7427	0.0604	-0.6754	74.1608	FALSE
3	18	20.903	0.8899	-16.5151	58.3212	FALSE
3	21	28.3114	0.4062	-9.1068	65.7295	FALSE
4	5	26.2702	0.552	-11.1479	63.6883	FALSE
4	6	15.7276	0.9941	-21.6906	53.1457	FALSE
4	7	17.2032	0.983	-20.2149	54.6213	FALSE
4	8	13.616	0.9998	-27.2105	54.4425	FALSE
4	10	14.1359	0.9996	-26.6906	54.9624	FALSE
4	11	22.0426	0.917	-18.7839	62.8692	FALSE
4	12	23.0055	0.882	-17.821	63.8321	FALSE
4	13	17.5998	0.9975	-27.1235	62.323	FALSE
4	15	14.6045	0.9977	-22.8136	52.0226	FALSE
3	16	-1.0533	1	-29.3388	27.2321	FALSE
4	16	-0.3813	1	-37.7994	37.0369	FALSE
4	17	14.9199	0.997	-22.4982	52.338	FALSE
4	18	21.5751	0.9715	-23.1481	66.2983	FALSE
4	20	32.0077	0.1978	-5.4105	69.4258	FALSE
4	21	28.9834	0.6983	-15.7398	73.7066	FALSE
5	6	-10.5426	0.9988	-38.8281	17.7428	FALSE
5	7	-9.067	0.9999	-37.3525	19.2184	FALSE
5	8	-12.6542	0.9979	-45.3154	20.007	FALSE
5	9	14.0709	0.961	-14.2145	42.3564	FALSE
5	10	-12.1343	0.9988	-44.7955	20.5269	FALSE
3	17	14.2478	0.956	-14.0376	42.5333	FALSE
3	15	13.9325	0.9647	-14.353	42.2179	FALSE
5	12	-3.2647	1	-35.9259	29.3966	FALSE
1	22	12.8247	0.9854	-15.4607	41.1102	FALSE
1	5	-25.558	0.13	-53.8434	2.7275	FALSE
1	9	-11.487	0.9962	-39.7725	16.7984	FALSE
1	11	-29.7855	0.1203	-62.4468	2.8757	FALSE
1	12	-28.8226	0.157	-61.4838	3.8386	FALSE
1	13	-34.2284	0.1172	-71.6465	3.1897	FALSE

1	14	-15.0855	0.9254	-43.3709	13.2	FALSE
1	18	-30.2531	0.2854	-67.6712	7.1651	FALSE
1	19	-12.5073	0.9891	-40.7927	15.7782	FALSE
1	20	-19.8205	0.5557	-48.106	8.4649	FALSE
1	21	-22.8448	0.7898	-60.2629	14.5734	FALSE
1	23	12.5431	0.9887	-15.7423	40.8286	FALSE
3	13	16.9277	0.9858	-20.4904	54.3458	FALSE
2	22	-5.8541	1	-34.1396	22.4313	FALSE
2	23	-6.1357	1	-34.4212	22.1497	FALSE
3	4	-0.6721	1	-38.0902	36.7461	FALSE
3	5	25.5981	0.1283	-2.6873	53.8836	FALSE
3	6	15.0555	0.9267	-13.2299	43.3409	FALSE
3	7	16.5311	0.8445	-11.7543	44.8166	FALSE
3	8	12.9439	0.9972	-19.7173	45.6052	FALSE
3	10	13.4638	0.9954	-19.1974	46.1251	FALSE
3	11	21.3706	0.6819	-11.2906	54.0318	FALSE
3	12	22.3335	0.6023	-10.3277	54.9947	FALSE
5	11	-4.2276	1	-36.8888	28.4337	FALSE
5	13	-8.6704	1	-46.0886	28.7477	FALSE
9	10	-26.2052	0.2985	-58.8665	6.456	FALSE
8	10	0.5199	1	-35.9965	37.0363	FALSE
7	12	5.8024	1	-26.8589	38.4636	FALSE
7	13	0.3966	1	-37.0215	37.8147	FALSE
7	14	19.5395	0.5831	-8.7459	47.825	FALSE
7	15	-2.5987	1	-30.8841	25.6868	FALSE
7	16	-17.5844	0.764	-45.8699	10.701	FALSE
7	18	4.3719	1	-33.0462	41.7901	FALSE
7	19	22.1177	0.3444	-6.1678	50.4031	FALSE
7	20	14.8045	0.9369	-13.481	43.0899	FALSE
7	21	11.7802	0.9999	-25.6379	49.1984	FALSE
8	9	26.7251	0.2654	-5.9361	59.3864	FALSE
8	11	8.4266	1	-28.0897	44.943	FALSE
7	10	-3.0673	1	-35.7285	29.5939	FALSE
8	12	9.3895	1	-27.1268	45.9059	FALSE
8	13	3.9838	1	-36.8428	44.8103	FALSE
8	14	23.1267	0.5355	-9.5345	55.7879	FALSE
8	15	0.9885	1	-31.6727	33.6497	FALSE
8	16	-13.9973	0.9925	-46.6585	18.664	FALSE
8	17	1.3039	1	-31.3573	33.9651	FALSE
8	18	7.9591	1	-32.8674	48.7856	FALSE
8	19	25.7049	0.3327	-6.9563	58.3661	FALSE
8	20	18.3917	0.8827	-14.2696	51.0529	FALSE
8	21	15.3674	0.9986	-25.4591	56.1939	FALSE

7	11	4.8394	1	-27.8218	37.5007	FALSE
7	9	23.138	0.2659	-5.1475	51.4234	FALSE
5	14	10.4725	0.9989	-17.8129	38.7579	FALSE
6	10	-1.5917	1	-34.2529	31.0696	FALSE
5	15	-11.6657	0.9953	-39.9511	16.6198	FALSE
5	16	-26.6515	0.0902	-54.9369	1.634	FALSE
5	17	-11.3503	0.9967	-39.6358	16.9351	FALSE
5	18	-4.6951	1	-42.1132	32.723	FALSE
5	19	13.0507	0.9823	-15.2348	41.3361	FALSE
5	20	5.7375	1	-22.548	34.0229	FALSE
5	21	2.7132	1	-34.7049	40.1313	FALSE
6	7	1.4756	1	-26.8098	29.7611	FALSE
6	8	-2.1116	1	-34.7728	30.5497	FALSE
6	9	24.6136	0.1746	-3.6719	52.899	FALSE
6	11	6.3151	1	-26.3461	38.9763	FALSE
7	8	-3.5872	1	-36.2484	29.074	FALSE
6	12	7.278	1	-25.3832	39.9392	FALSE
6	13	1.8722	1	-35.5459	39.2903	FALSE
6	14	21.0151	0.4415	-7.2703	49.3006	FALSE
6	15	-1.123	1	-29.4085	27.1624	FALSE
6	16	-16.1088	0.8719	-44.3943	12.1766	FALSE
6	17	-0.8077	1	-29.0931	27.4778	FALSE
6	18	5.8476	1	-31.5706	43.2657	FALSE
6	19	23.5933	0.2349	-4.6921	51.8788	FALSE
6	20	16.2801	0.8612	-12.0053	44.5656	FALSE
6	21	13.2559	0.9994	-24.1623	50.674	FALSE
22	23	-0.2816	1	-28.567	28.0039	FALSE

Scanning Electron Microscopy (SEM)

SEM images with submicron resolution did not show evidence of an effect of processing on microstructure or surface morphology (Figure S2). Additionally, SEM images did not provide insight into interfacial adhesion between PLA and PBAT. Very high magnifications (i.e. > x20000, or sometimes even x10000) were not achievable due to the electron probe damaging the film surface during scanning. Voltages between 0.5 and 5.0 kV were explored to avoid sample damage, which was unavoidable at high magnifications irrespective of the voltage used (see Supporting Information Figure S2(m-n)).

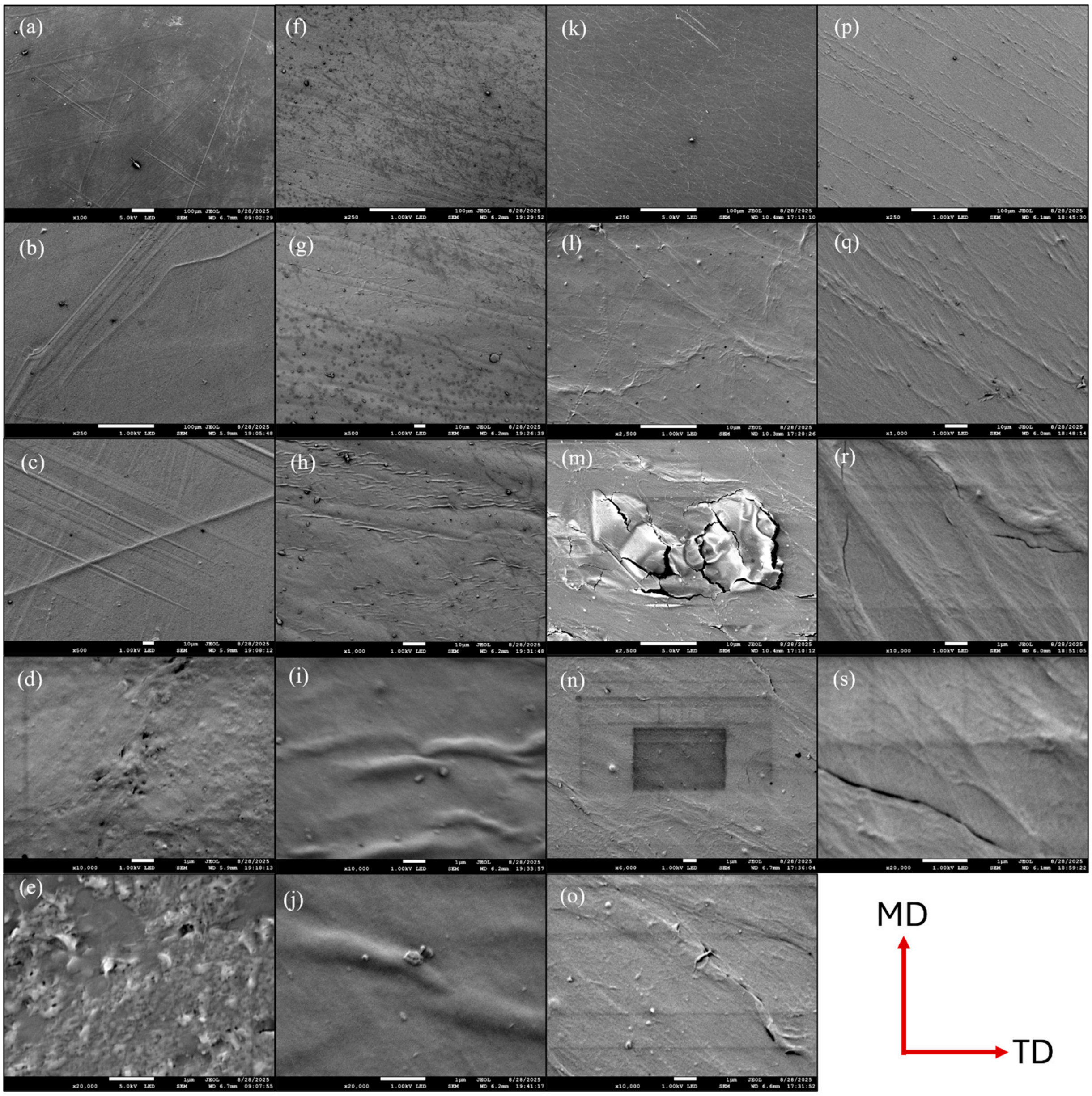

Figure S2. SEM images at various magnifications for (a-e) pressed 10%/90% PLA/PBAT (17[C]), (f-j) 90%/10% PLA/PBAT λ 5×5 90°C (2[D]), (k-o) 50%/50% PLA/PBAT λ 5×5 90°C (7[D]), and (p-s) pure PLA λ 5×5 90°C (11[D]). The machine direction (MD) and transverse direction (TD) of stretching for biaxially stretched samples are labeled in the bottom right. MD and TD were maintained for all photos.

Thermal Analyses for Validation Dataset

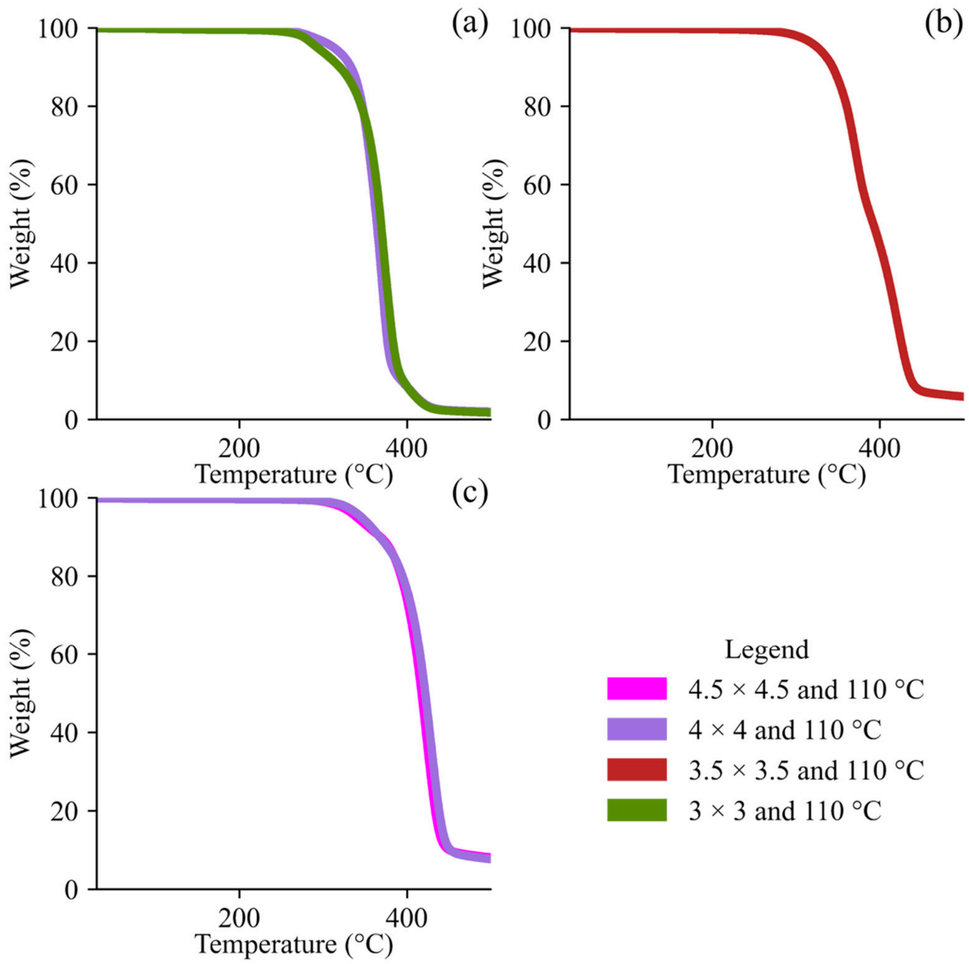

Figure S3. TGA thermograms for runs 19^V, 20^V, 21^V, 22^V, and 23^V organized by composition as in: (a) 90%/10% PLA/PBAT, (b) 50%/50% PLA/PBAT, and (c) 10%/90% PLA/PBAT.

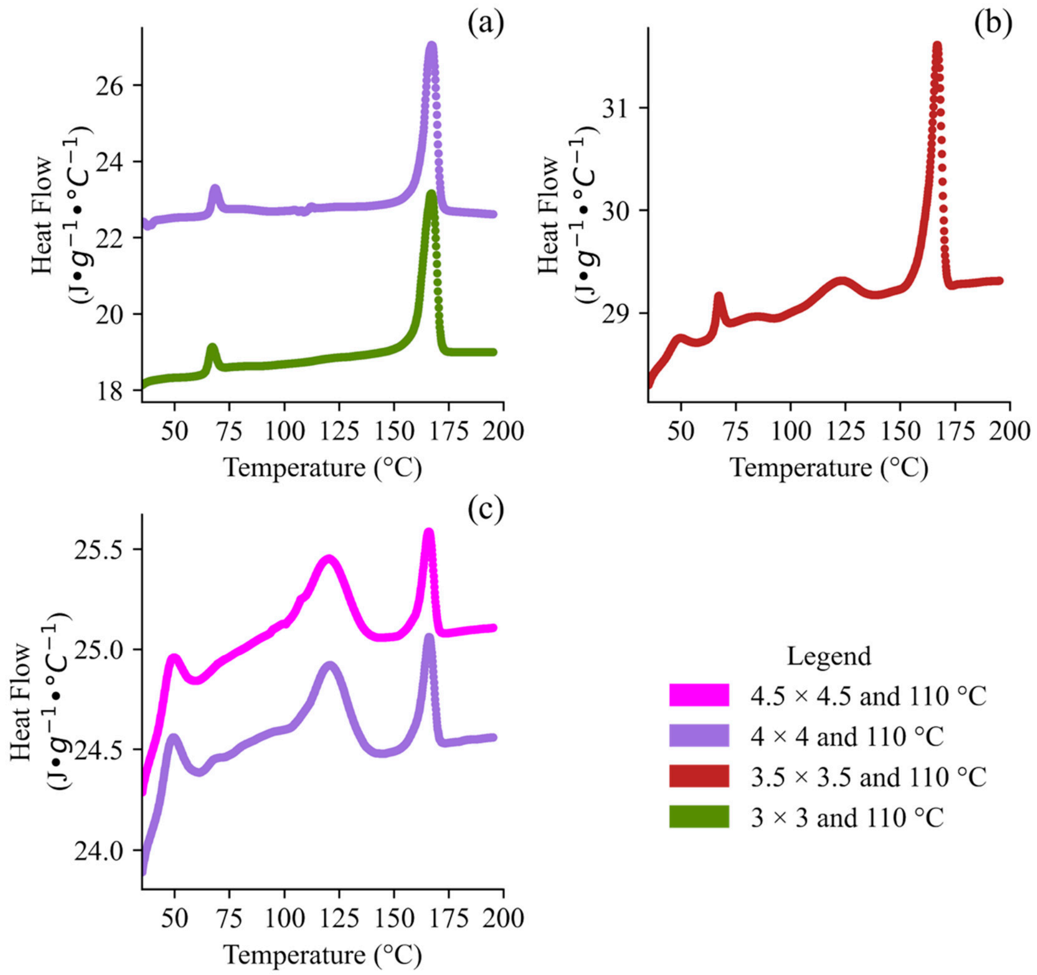

Figure S4. DSC thermograms, Endo up, for first heating scan of runs 19^V, 20^V, 21^V, 22^V, and 23^V organized by composition as in: (a) 90%/10% PLA/PBAT, (b) 50%/50% PLA/PBAT, and (c) 10%/90% PLA/PBAT.

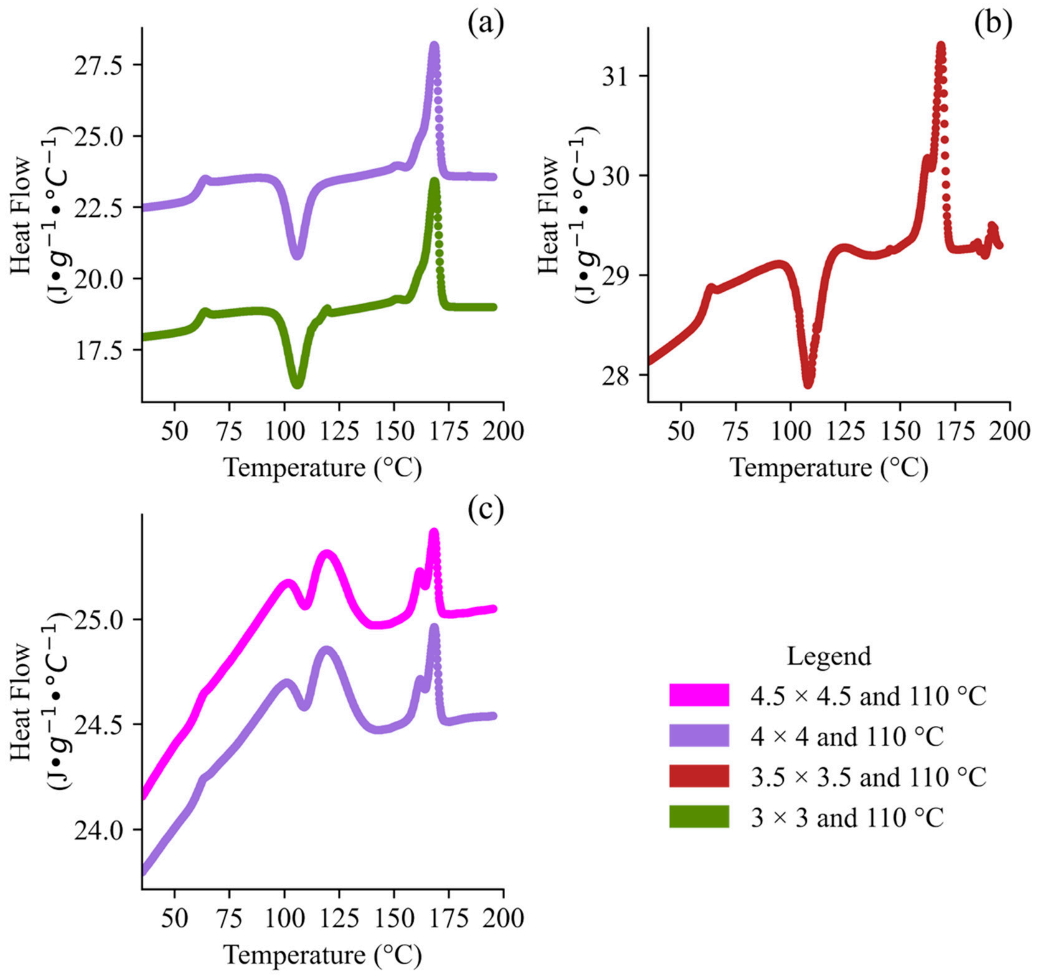

Figure S5. DSC thermograms, Endo up, for second heating scan of runs 19^V, 20^V, 21^V, 22^V, and 23^V organized by composition as in: (a) 90%/10% PLA/PBAT, (b) 50%/50% PLA/PBAT, and (c) 10%/90% PLA/PBAT.

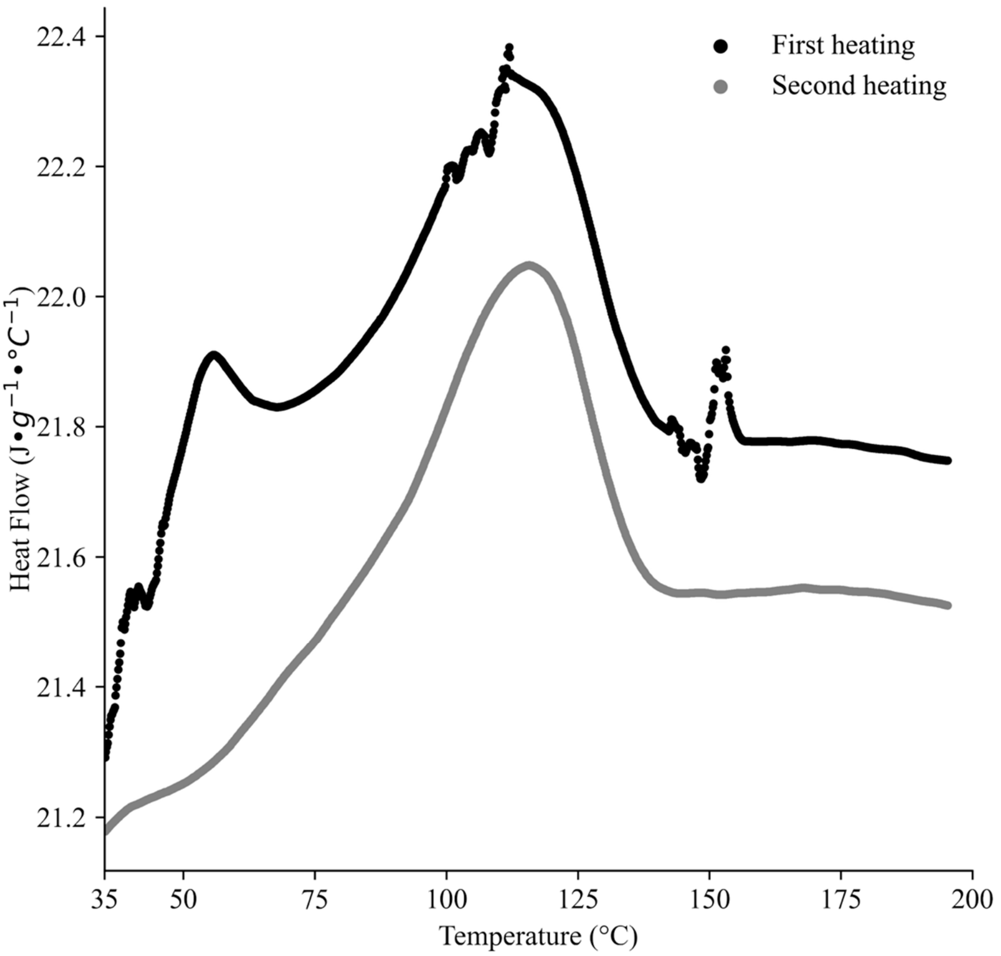

Figure S6. DSC thermogram, Endo up, for first (black series) and second (grey series) heating scans of commercial PBAT resin.

References

1. ASTM Standard D 638-22: Standard Test Method for Tensile Properties of Plastics. ASTM International: West Conshohocken, PA, USA, 2022.
